# Divergent Tandem Acyl Carrier Proteins Necessitate In-Series Polyketide Processing in the Leinamycin Family

**DOI:** 10.1002/anie.202414165

**Published:** 2024-11-06

**Authors:** Annabel P. Phillips, Ashley J. Winter, Chloe M. Hooper, Christopher Williams, John Crosby, Christine L. Willis, Matthew P. Crump

**Affiliations:** School of Chemistry, University of Bristol, Bristol, BS8 1TS (UK)

**Keywords:** β-Branching, Biosynthesis, Leinamycin, Polyketides, Weishanmycin

## Abstract

The leinamycin family of polyketides are promising antitumor antibiotics, yet several aspects of their biosynthesis remain elusive. All leinamycin family members bear a sulfur-containing moiety which is essential for the anticancer activity exhibited by leinamycin. The key building blocks required for the incorporation of these functionalities are introduced in the final module of the polyketide synthase (PKS), which elegantly combines β-branching and thiocysteine incorporation to generate a diverse library of sulfur-based molecular scaffolds. Two acyl carrier proteins (ACPs) form a key didomain component of this module, but their amino acid sequence divergence has brought into question the common notion of functional equivalence. Here, we provide unprecedented functional evidence that these tandem ACPs play distinct roles in the final module of polyketide assembly. Using the weishanmycin biosynthetic pathway as a template, the in vitro reconstitution of key polyketide chain extension and β-branching steps in this module has revealed strict functional selectivity for a single ACP. Furthermore, we propose a cryptic transacylation step must occur prior to polyketide off-loading and cyclization. Altogether, these mechanistic investigations suggest that an atypical inseries mechanism underpins sulfur incorporation in the leinamycin family, and provides significant progress towards delineating their late-stage assembly.

## Introduction

Polyketides are an extensive class of secondary metabolites that exhibit a range of biological activities, and therefore have important applications in drug discovery and development.^[1]^ They are commonly manufactured by type I polyketide synthases (PKSs): megadalton, multimodular enzyme assemblies that biosynthesize polyketides through the repeated incorporation of two carbon ‘ketide’ units derived from acyl-coenzyme A (CoA) building blocks.^[2]^ Each module contains the necessary enzymatic domains for a round of polyketide chain elongation via decarboxylative Claisen condensation (DCC), catalyzed by ketosynthases (KSs), and β-keto processing. The catalytic domains act on acyl carrier protein (ACP)-bound intermediates that are tethered via a flexible phosphopantetheine (Ppant) arm and are primed with acyl extender units by acyltransferases (ATs). While type I *cis*-AT PKSs harbour one dedicated AT per module, *trans*-AT systems have one or more discrete ATs that interact with ACPs *in-trans*. In comparison to their *cis*-AT counterparts, *trans*-AT PKSs have tolerated a more diverse combination of evolutionary events, and inherently catalyze more unusual biosynthetic transformations, encode unique domains, and frequently employ *trans*-acting enzymes.^[3]^ Coupled with an underlying modular architecture, this makes *trans*-AT PKSs exemplary candidates for PKS engineering to produce novel bioactive molecules.^[4]^

**Figure 1:**
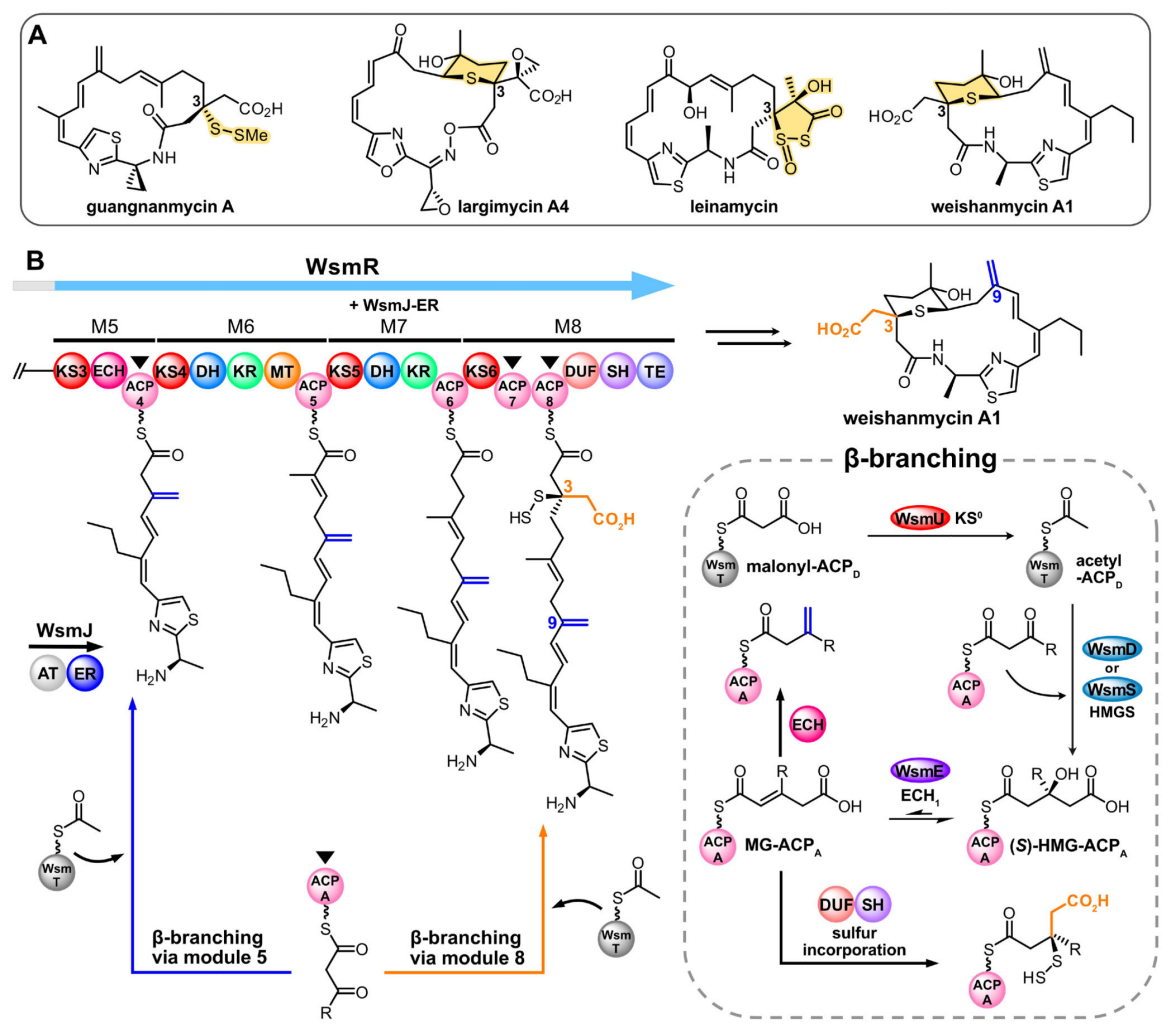
A) Structures of key members of the leinamycin family of polyketides. Their C-3 sulfur functionalities are highlighted. B) The WsmR PKS modules in the proposed weishanmycin biosynthetic pathway. ACPs and KSs are numbered in sequential order according to the NRPS/PKS assembly line. ACPs are primed with malonyl units by WsmJ, an acyltransferase/enoylreductase (AT/ER) didomain. β-Branching via module 5 produces the C-9 *exo*-β-methylene branch (blue), whereas β-branching via module 8 furnishes a carboxylated β-branch at C-3 (orange). Both β-branching modules recruit acetyl-WsmT. Black triangles mark the putative ACP_A_s. β-Branching in module 8 is followed by incorporation of a hydropersulfide moiety by successive recruitment of the domain of unknown function (DUF) and thiocysteine lyase (SH). After polyketide off-loading, several transformations under oxidative conditions are believed to result in the major metabolite, weishanmycin A1.^[15]^ A full biosynthetic pathway is shown in Scheme S1. DH: dehydratase; KR: ketoreductase; MT: methyltransferase.

β-Branching is a key means of structural diversification in polyketide biosynthesis and most commonly occurs in *trans*-AT PKSs.^[5]^ A set of *trans*-acting proteins, known collectively as a 3-hydroxy-3-methylglutaryl synthase (HMGS) cassette, are responsible for modifying the electrophilic β-ketone post-DCC while the polyketide intermediate is bound to a modular ACP that is termed the acceptor (ACP_A_).^[6]^ A minimal HMGS cassette contains: a donor ACP (ACP_D_), a decarboxylating, non-elongating KS (lacking the catalytic Cys residue required for condensation; KS^0^) or acyltransferase/decarboxylase didomain (AT/DC), an HMGS, and a dehydrating enoyl-CoA hydratase (ECH_1_). A typical β-branching reaction sequence begins with decarboxylation of a malonyl or methylmalonyl unit by a KS^0^ or bifunctional AT/DC,^[7]^ respectively, to produce acyl-ACP_D_ (Figure 1B). This undergoes aldol addition with an ACP_A_-bound β-ketothioester, which is catalyzed by the HMGS, to produce (*S*)-3-hydroxy-3-methylglutaryl (HMG)-ACP_A_. Subsequent dehydration by an ECH_1_ produces 3-methylglutaconyl (MG)-ACP_A_ in a reversible equilibrium.^[8]^ An optional decarboxylating ECH (ECH_2_) may act *in-trans* or *in-cis* to furnish an α,β- or β,γ-unsaturated β-branch, depending on its regioselectivity.^[9]^ Further modification can also result from additional tailoring enzymes.^[5a]^ The library of β-branches available via choice of HMGS cassette can therefore provide considerable diversification to polyketide structure.

To prevent erroneous β-branching, the HMGS must exhibit stringent selectivity for its cognate ACP_A_, yet molecular features that govern this are not fully understood.^[10]^ ACP_A_s are often arranged as tandem domains in the PKS module and canonically possess a diagnostic ‘Trp flag’ six residues downstream of the catalytic Ser residue.^[11]^ A few exceptions and ambiguities were identified, and several biosynthetic pathways are now known to utilize tandem ACPs in β-branching that lack this characteristic residue, including virginiamycin, ripostatin and leinamycin PKSs.^[12]^ Tandem ACPs also generally display a high sequence identity to each other, encode identical functions, and enhance the metabolic flux of the polyketide through the pathway.^[6,13]^ However, a recent study exploring the divergent roles of the tandem ACP pair in virginiamycin’s β-branching pathway, VirA ACP_5a/b_, has led to speculation regarding the assumed functional equivalence of other tandem ACPs.^[14]^

The leinamycin family of polyketides have promising potential as anticancer agents due to their sulfur-containing functionalities (Figure 1A). The unique 1,3-dioxo-1,2-dithiolane moiety in leinamycin for example, is essential for its antitumor activity.^[16]^ All leinamycin-type non-ribosomal peptide synthetase (NRPS)/PKS hybrids comprise a total of eight modules, with an identical domain architecture in their terminal off-loading module (module 8, KS-ACP-ACP-DUF-SH-TE) that universally installs a carboxylated β-branch and hydropersulfide moiety, respectively.^[17]^ It is assumed that the module 8 tandem ACPs act in-parallel (i.e., are functionally equivalent), but the absence of Trp flags means functional assignment of these ACPs is ambiguous.^[11]^ This necessitated further investigation, as it fundamentally underpins the mechanism of sulfur incorporation in this polyketide family.

Weishanmycin is a member of the leinamycin family that is isolated from *Streptomyces* sp. CB02120-2 and features a tetrahydrothiopyran ring embedded within its macrolactam core.^[15]^ Eight modules encoded over five open reading frames (ORFs) assemble the weishanmycin backbone (Scheme S1), two of which (WsmR modules 5 and 8) are β-branching modules. The HMGS cassette comprises WsmT (ACP_D_), WsmU (KS^0^), WsmE (ECH_1_) and two HMGSs, WsmD and WsmS. Phylogenetic analysis has suggested that WsmD and WsmS clade separately, and it is therefore proposed that the β-branching modules select for different HMGSs.^[15,18]^ Module 5 introduces a β,γ-unsaturated β-branch (referred to as an *exo*-β-methylene branch herein), with the alternate double bond stereochemistry controlled by the presence of an *in-cis* modular ECH_2_ (mECH) domain (Figure 1B).^[15,19]^ Conversely, the carboxylated β-branch is retained in module 8 as there is no *trans*-acting ECH_2_ encoded by the HMGS cassette (Figure 1B). Instead, conjugate addition of thiocysteine via the domain of unknown function (DUF) is proposed to occur. Subsequent C−S bond cleavage catalyzed by the PLP-dependent thiocysteine lyase (SH) furnishes the C-3 hydropersulfide moiety before the polyketide is cyclized by the module 8 thioesterase (TE) and converted to weishanmycin A1 (**Scheme S1**).^[15,17]^ We used the weishanmycin biosynthetic pathway as a model system to probe the roles and selectivity of the tandem ACPs (WsmR ACP7-8) and HMGS cassette components in-parallel.

Previous in vivo studies have been applied to leinamycin-type systems to interrogate β-branching pathways.^[18,20]^ To probe the molecular intricacies of the final stages of polyketide assembly, we chose an in vitro approach. Using purified protein components, polyketide intermediate mimics and mass spectrometry assays, we demonstrate that the didomain ACPs within module 8 exhibit divergent roles in the biosynthesis of leinamycin-type polyketides. Combined with bioinformatic analysis and site-directed mutagenesis, these studies have identified molecular recognition features that govern HMGS selectivity in module 8 and provide unprecedented mechanistic insights into the final stages of polyketide assembly in the leinamycin family.

## Results and Discussion

Investigations into the role of the WsmR ACP7-8 didomain began with sequence analysis and comparisons to analogous ACPs from the guangnanmycin (GnmT), largimycin (LrgJ) and leinamycin (LnmJ) PKSs. The canonical ACP_A_ Trp flag is absent in all eight ACPs analyzed and could therefore not be used as a diagnostic to identify putative ACP_A_s (Figure S1). Residues in equivalent positions (Ala, Leu, Thr and Val) are not conserved, suggesting that they may not play a direct role in controlling molecular recognition of HMGS cassette enzymes. Notably, these ACPs also lack significant sequence identity and similarity (27 % and 46 %, respectively, for WsmR ACP7-8) when compared to characterized functionally equivalent tandem ACP_A_s from the kalimantacin and mupirocin biosynthetic pathways (Figure S1).^[9,11]^ This led us to the hypothesis that functional equivalence and in-parallel polyketide chain processing within module 8 was unlikely.

Previous bioinformatic studies have shown that β-branching ACP_A_s clade separately from non-ACP_A_s.^[11]^ We decided to utilize this approach to determine if the sequence discrepancy between these module 8 tandem ACPs was observed within the wider leinamycin family. Bioinformatic analysis of 58 ACPs from 29 leinamycin-type PKSs revealed that all of the analyzed ACPs clade according to their intramodular location, with the first ACP of a tandem pair clading separately from the second ACP (referred to as clade 1 and clade 2, respectively) (Figure S2). This suggested that the sequence divergence amongst the module 8 ACP pairs in leinamycin-type PKSs is universal, and additionally strengthens the hypothesis that they may have functionally distinct roles in the biosynthesis of this family of polyketides.

To determine if both of the WsmR tandem ACPs could be malonylated, WsmR ACP7 and WsmR ACP8 were overproduced in *E. coli* and purified to homogeneity as soluble excised domains (Figure S3). Both ACPs were confirmed to be folded and monomeric in solution by analytical size exclusion chromatography (SEC) and ^1^H-^15^N heteronuclear single quantum coherence (HSQC) NMR analysis (Figure S3). The *trans*-acting AT, WsmJ, could not be acquired as a soluble enzyme. We therefore overproduced and purified LnmG, an AT homolog (53 % sequence identity shared with WsmJ), as previously described.^[21]^ After conversion of the *apo*-ACPs to their *holo* derivatives by incubation with CoA and MupN, a phosphopantetheinyl transferase, the ability of LnmG to malonylate the *holo*-ACPs was assessed by electrospray mass spectrometry (ESMS). *Holo*-WsmR ACP7 and *holo*-WsmR ACP8 were individually incubated with LnmG and malonyl-CoA, and ESMS analysis confirmed that both ACPs were recognized and malonylated by the surrogate AT (Figure S4). Ppant ejection assays via collision-induced dissociation of the derivatized Ppant arm yielded characteristic fragmentation ions with high mass accuracy that verified the presence of ACP-bound malonyl species in both cases (Figure S4).^[22]^ Both ACPs were therefore functional with regards to malonylation and should be available for subsequent DCC.

**Figure 2:**
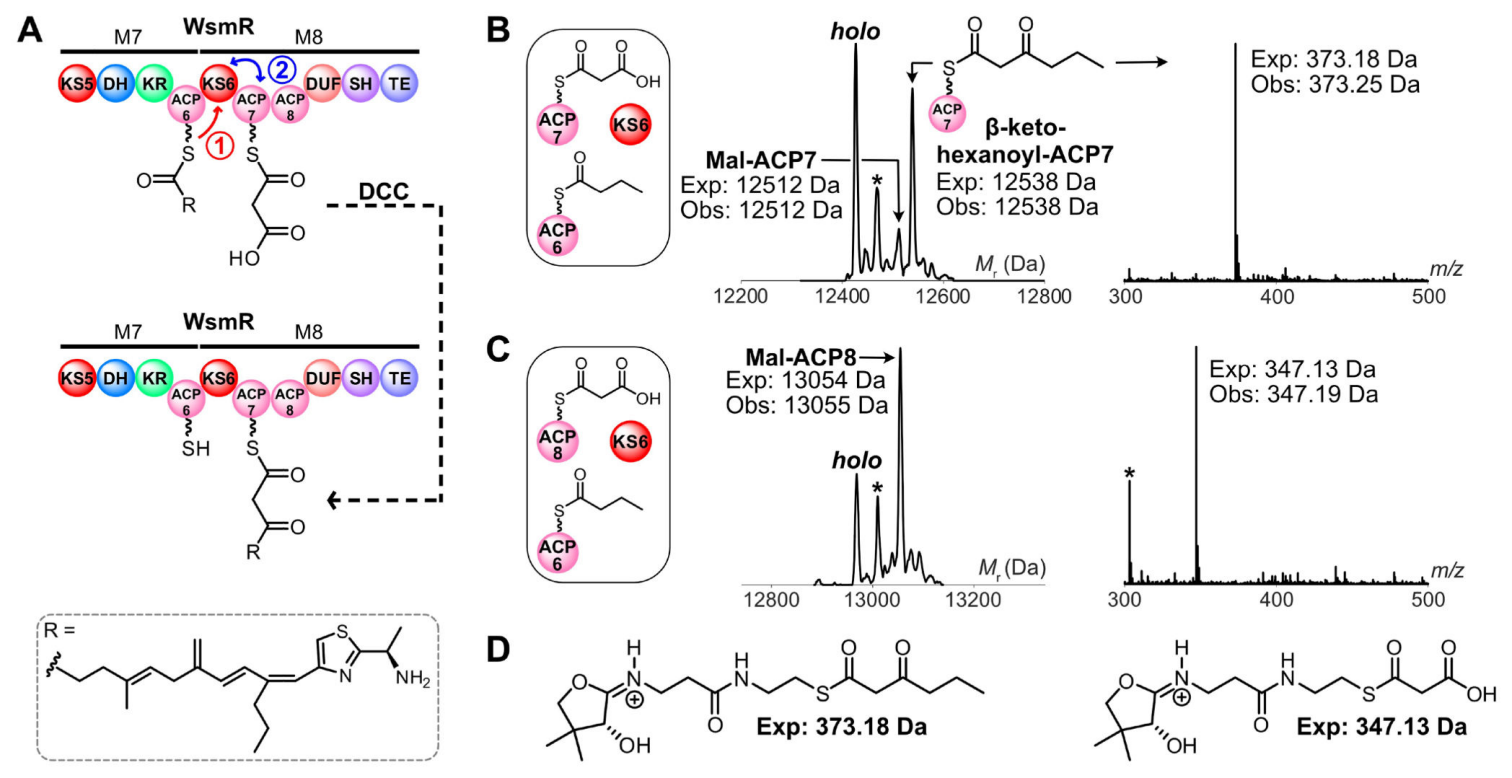
Decarboxylative Claisen condensation (DCC) reaction scheme and ESMS assays. A) Proposed reaction scheme for DCC in WsmR module 8 based on ESMS assays. B) Deconvoluted spectrum and corresponding Ppant ejection for DCC assay with malonyl (Mal)-WsmR ACP7. C) Deconvoluted spectrum and corresponding Ppant ejection for DCC assay with malonyl (Mal)-WsmR ACP8. D) Expected Ppant ejection ions. *=acetyl species.

With the module 8 didomain ACPs malonylated, we next determined if the preceding KS, WsmR KS6, could catalyze DCC-derived chain extension in collaboration with both ACPs (Figure 2A). The gene encoding WsmR KS6 was therefore cloned and recombinantly expressed. The resulting protein was purified to homogeneity and confirmed to be dimeric by analytical SEC, which is characteristic of elongating KSs (Figure S5).^[23]^ To improve the reaction efficiency, we sought to create an extended tri-component assay that included the preceding ACP, WsmR ACP6, to relay an acyl substrate to WsmR KS6 and prime it for DCC. WsmR ACP6 was therefore overproduced and purified as a discrete monomeric domain (Figure S5). A butyryl group was used as a surrogate for the native polyketide chain and was transferred onto *apo*-WsmR ACP6 using butyryl-CoA and MupN (Figure S6). Incubation of butyryl-WsmR ACP6, malonyl-WsmR ACP7 and WsmR KS6 led to production of the DCC product, β-keto-hexanoyl-WsmR ACP7, as confirmed by ESMS (exp: 12538 Da, obs: 12538 Da) and Ppant ejection (exp: 373.18 Da, obs: 373.25 Da) (Figure 2B). Conversely, when malonyl-WsmR ACP7 was replaced by malonyl-WsmR ACP8, DCC did not occur (exp: 13080 Da, not observed) (Figure 2C). Substitution of malonyl-WsmR ACP7 for non-cognate malonyl-WsmR ACP4, the module 5 ACP_A_ candidate, also gave no observable DCC product (Figure S6). Intriguingly, these assays indicated that despite malonylation of WsmR ACP8, only WsmR ACP7 can participate in DCC with WsmR KS6. The additional assay with malonyl-WsmR ACP4 further supports the premise that the lack of DCC in the presence of malonyl-WsmR ACP8 is attributable to strict KS specificity. Such specific ACP/KS protein-protein interactions are essential to avoid ‘module skipping’ and maintain biosynthetic fidelity.^[24]^ The WsmR ACP7/WsmR KS6 specificity implied that WsmR ACP7 may be the sole module 8 ACP required for β-branching.

**Figure 3:**
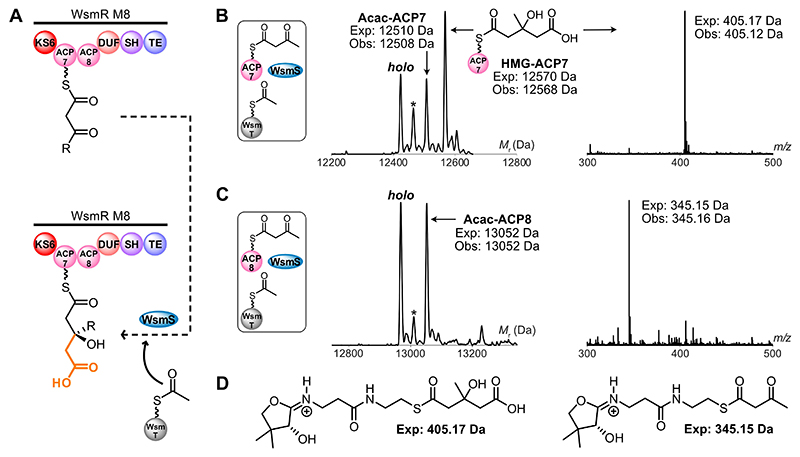
WsmS-catalyzed aldol addition reaction scheme and ESMS assays. A) Proposed reaction scheme for the key β-branching step in WsmR module 8. B) Deconvoluted spectrum and corresponding Ppant ejection for WsmS assay with acetoacetyl (Acac)-WsmR ACP7. C) Deconvoluted spectrum and corresponding Ppant ejection for WsmS assay with acetoacetyl (Acac)-WsmR ACP8. D) Expected Ppant ejection ions. * =acetyl species.

Following DCC, aldol addition between acetyl-WsmT (ACP_D_) and the ACP_A_-bound β-ketothioester should occur to produce the HMG-ACP_A_ intermediate (Figure 3A). To interrogate this reaction and HMGS selectivity in-parallel, genes encoding WsmD and WsmS (HMGSs), and the ACP_D_, WsmT, were cloned, overexpressed and the resulting proteins purified (Figure S3, S7). Analytical SEC suggested that both HMGSs were dimeric in solution, while WsmT existed as a monomer (Figure S3, S7). Acetyl-pantetheine was enzymatically converted to derivatize WsmT, while the putative ACP_A_s, WsmR ACP4, ACP7 and ACP8, were derivatized with acetoacetyl-pantetheine as a β-ketothioester mimic (Figure S8). Initially, the selectivity of WsmD was assayed by incubating with acetyl-WsmT and acetoacetyl-WsmR ACP4. ESMS analysis and Ppant ejection confirmed formation of HMG-WsmR ACP4 (Figure S9). However, substitution of WsmD for WsmS abrogated HMG-WsmR ACP4 production, confirming that β-branch incorporation in module 5 is exclusively controlled by WsmD (Figure S9). Conversely, when acetoacetyl-WsmR ACP4 was substituted for acetoacetyl-derivatized WsmR ACP7 or ACP8 in WsmD assays, neither HMG-WsmR ACP7 nor HMG-WsmR ACP8 was observed by ESMS (Figure S10). Together, this confirmed a strict pairing between WsmD and WsmR ACP4 within module 5.

Next, WsmS was incubated with acetyl-WsmT and acetoacetyl-WsmR ACP7 and the reaction monitored by ESMS. A new ACP7-bound species was observed with a mass consistent with HMG-WsmR ACP7 (exp: 12570 Da, obs: 12568 Da) (Figure 3B). Production of HMG-WsmR ACP7 was verified by Ppant ejection, which generated a characteristic HMG-Ppant ion (exp: 405.17 Da, obs: 405.12 Da). The analogous assay with acetoacetyl-WsmR ACP8, however, failed to generate HMG-WsmR ACP8 (exp: 13112 Da, not observed), instead propagating *holo*-WsmR ACP8 according to ESMS analysis (Figure 3C). This confirmed that WsmS initiates β-branching in module 8 but is only permitted via interaction with WsmR ACP7, providing unprecedented in vitro evidence for tandem ACP functional inequivalence in polyketide biosynthesis.

**Figure 4:**
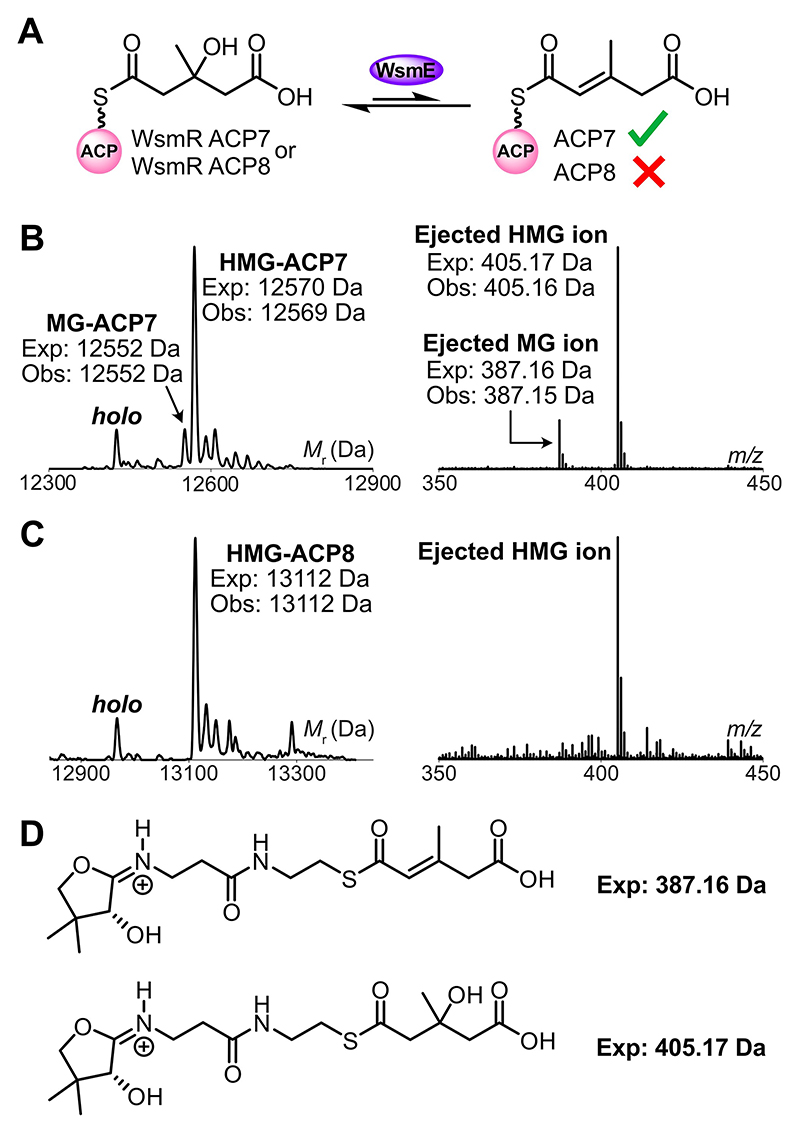
WsmE ESMS assays. A) Proposed reaction scheme for WsmE-catalyzed dehydration of HMG-WsmR ACP7. Dehydration of HMG-WsmR ACP8 was not observed. B) Deconvoluted spectrum and corresponding Ppant ejection of WsmE assay with HMG-WsmR ACP7. C) Deconvoluted spectrum and corresponding Ppant ejection of WsmE assay with HMG-WsmR ACP8. D) Expected Ppant ejection ions.

If the module 8 tandem ACPs are truly inequivalent, subsequent WsmE-catalyzed dehydration of HMG-ACP_A_ to produce the nascent MG-ACP_A_ species should only occur on WsmR ACP7 (Figure 4A). Due to the transient nature of MG-ACP_A_, we reconstituted this step with WsmR ACP7 and WsmR ACP8 derivatized with (*R,S*)-HMG-CoA (Figure S11). Incubation of HMG-WsmR ACP7 with WsmE (ECH_1_), which was overproduced and purified as a trimer (Figure S7), revealed that WsmE could recognize WsmR ACP7 to produce the transient MG-WsmR ACP7 species. This was confirmed by ESMS analysis (exp: 12552 Da, obs: 12552 Da) and Ppant ejection, which yielded an MG-bound fragment ion (exp: 387.16 Da, obs: 387.15 Da) (Figure 4B).

MG-WsmR ACP4 was also observed by ESMS when equivalent WsmE assays were carried out with HMG-WsmR ACP4, verifying that WsmE as the only ECH_1_ can function across both modules involved in β-branching (Figure S9). HMG-WsmR ACP8 failed to yield MG-WsmR ACP8 (exp: 13094 Da, not observed) in the presence of WsmE, confirming that it does not participate in any β-branching step (Figure 4C).

**Figure 5:**
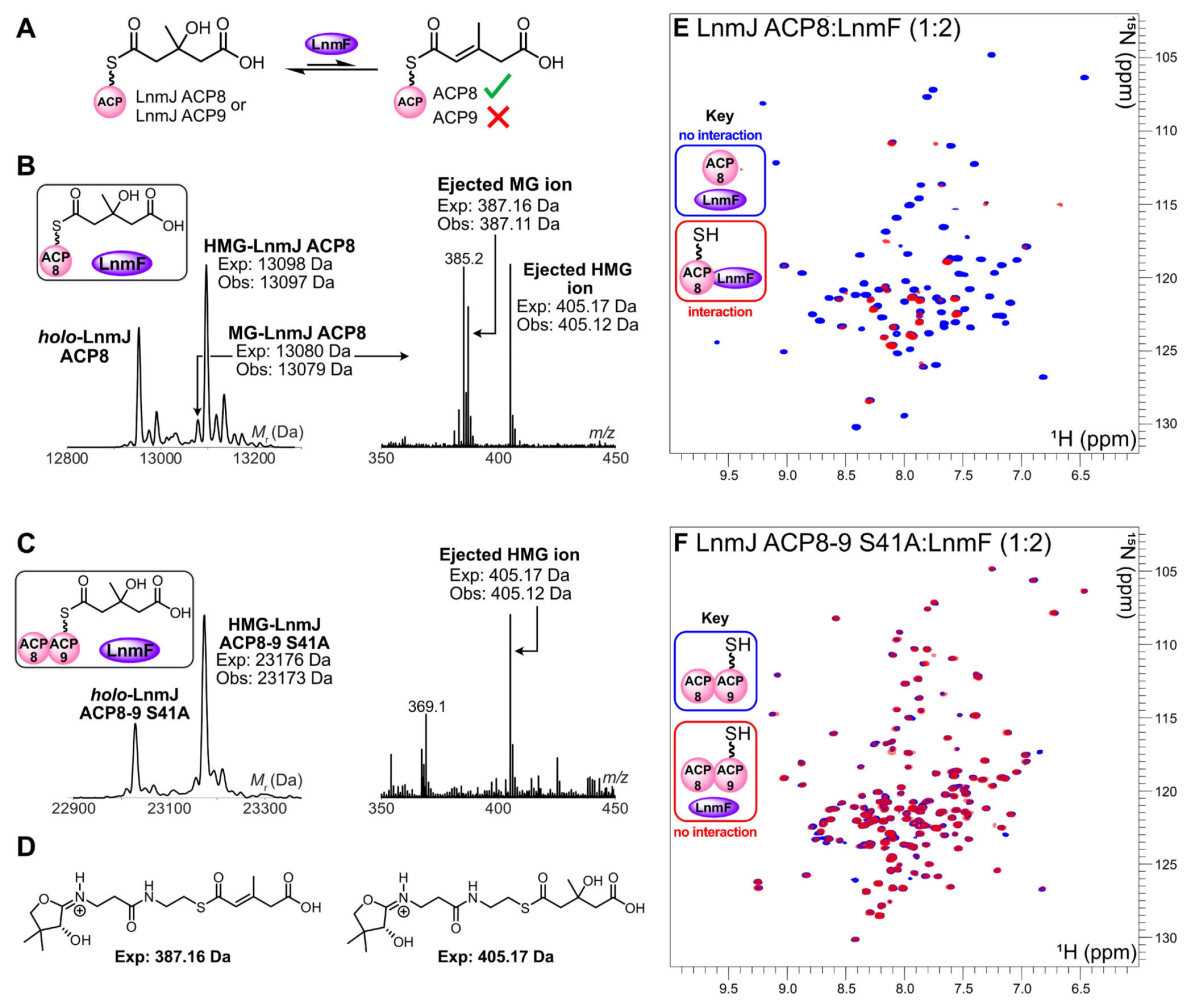
LnmF ESMS assays and NMR titrations. A) Proposed reaction scheme for LnmF-catalyzed dehydration of HMG-LnmJ ACP8. Dehydration of HMG-LnmJ ACP9 was not observed. B) Deconvoluted spectrum and corresponding Ppant ejection of LnmF assay with HMG-LnmJ ACP8. Additional Ppant ion at 385.20 Da was attributed to fragmentation of the ACP (Figure S13). C) Deconvoluted spectrum and corresponding Ppant ejection of LnmF assay with HMG-LnmJ ACP8-9 S41A. D) Expected Ppant ejection ions. E) Overlay of ^1^H-^15^N HSQCs of ^15^N *apo*-LnmJ ACP8 in the presence of LnmF in two-fold excess (blue) and ^15^N *holo*-LnmJ ACP8 in the presence of LnmF in two-fold excess (red). F) Overlay of ^1^H-^15^N HSQCs of ^15^N *holo*-LnmJ ACP8-9 S41A in the absence (blue) and presence (red) of LnmF in two-fold excess. Full ^1^H-^15^N HSQC data is shown in Figure S14.

To further generalize the observation of functional inequivalence, we next turned our attention to the leinamycin biosynthetic pathway. The homologous didomain ACPs from the LnmJ PKS (ACP8 and ACP9) and the *trans*-acting ECH_1_ (LnmF), were overproduced and purified to homogeneity (Figure S12). LnmJ ACP8 and LnmF were isolated as soluble proteins and confirmed to be monomeric and trimeric, respectively, by analytical SEC (Figure S12). LnmJ ACP9 did not fold as a single domain according to SEC, but expression of a didomain ACP8-9 construct in which the conserved ACP8 Ser residue was mutated to an Ala residue (LnmJ ACP8-9 S41A according to the excised domain sequence, or S6088A in the native PKS context) gave folded monomeric protein as judged by analytical SEC (Figure S12). LnmJ ACP8 and LnmJ ACP8-9 S41A were recognized by LnmG and malonylated in vitro (Figure S12), supporting the evidence that the module 8 didomain ACPs were folded and reinforcing previous malonylation data acquired for LnmJ ACP8.^[21]^ Following derivatization with (*R,S*)-HMG-CoA, the ACPs were then individually incubated with LnmF (Figure 5A, Figure S13). MG-LnmJ ACP8 was successfully observed by ESMS (exp: 13080 Da, obs: 13079 Da) and the corresponding MG-Ppant ion generated by Ppant ejection (exp: 387.16 Da, obs: 387.11 Da) (Figure 5B). A deconvoluted signal corresponding to MG-LnmJ ACP8-9 S41A was not observed (exp: 23158 Da) (Figure 5C).

Furthermore, we acquired ^1^H-^15^N HSQC spectra of ^15^N-labeled *apo*-LnmJ ACP8, *holo*-LnmJ ACP8 and *holo*-LnmJ ACP8-9 S41A and monitored spectral changes upon addition of unlabeled LnmF in two-fold excess (Figure 5E-F, Figure S14). There were no discernible chemical shift perturbations (CSPs) or resonance broadening when ^1^H-^15^N HSQCs of ^15^N *apo*-LnmJ ACP8 in the absence and presence of LnmF were compared (Figure 5E). However, significant resonance broadening suggested formation of a ^15^N *holo*-LnmJ ACP8/LnmF complex (Figure 5E), highlighting the importance of Ppant arm recognition for ECH_1_ recruitment.^[14]^ When LnmF was incubated with ^15^N *holo*-LnmJ ACP8-9 S41A, no CSPs or resonance broadening were observed (Figure 5F), proving that there is no interaction with either *apo*-LnmJ ACP8 or *holo*-LnmJ ACP9. To demonstrate that the S41A mutation had no impact on the structure of LnmJ ACP8-9, we generated wild-type LnmJ ACP8-9 via site-directed mutagenesis, which was overproduced and purified as a monomeric species (Figure S15). Comparison of ^1^H-^15^N HSQCs obtained for ^15^N *apo*-LnmJ ACP8-9 and its S41A mutant indicated that resonances correlating to the backbone amides overlayed with negligible CSPs, suggesting that there were no structural alterations (Figure S15). Comparisons of the HSQCs for both ^15^N LnmJ ACP8-9 constructs to the HSQC obtained for ^15^N *apo*-LnmJ ACP8 also suggested that there were no observable structural differences for LnmJ ACP8 as an excised domain compared to its didomain variants (Figure S15). The absence of structural perturbations suggested that the S41A mutation had not affected the function of LnmJ ACP9, and that the secondary structure of the excised versus tandem ACPs were equivalent. Coupled with the weishanmycin β-branching assays and phylogenetic analysis, these results suggested that the divergent roles of the module 8 ACP didomains may be universal to the leinamycin family.

**Figure 6:**
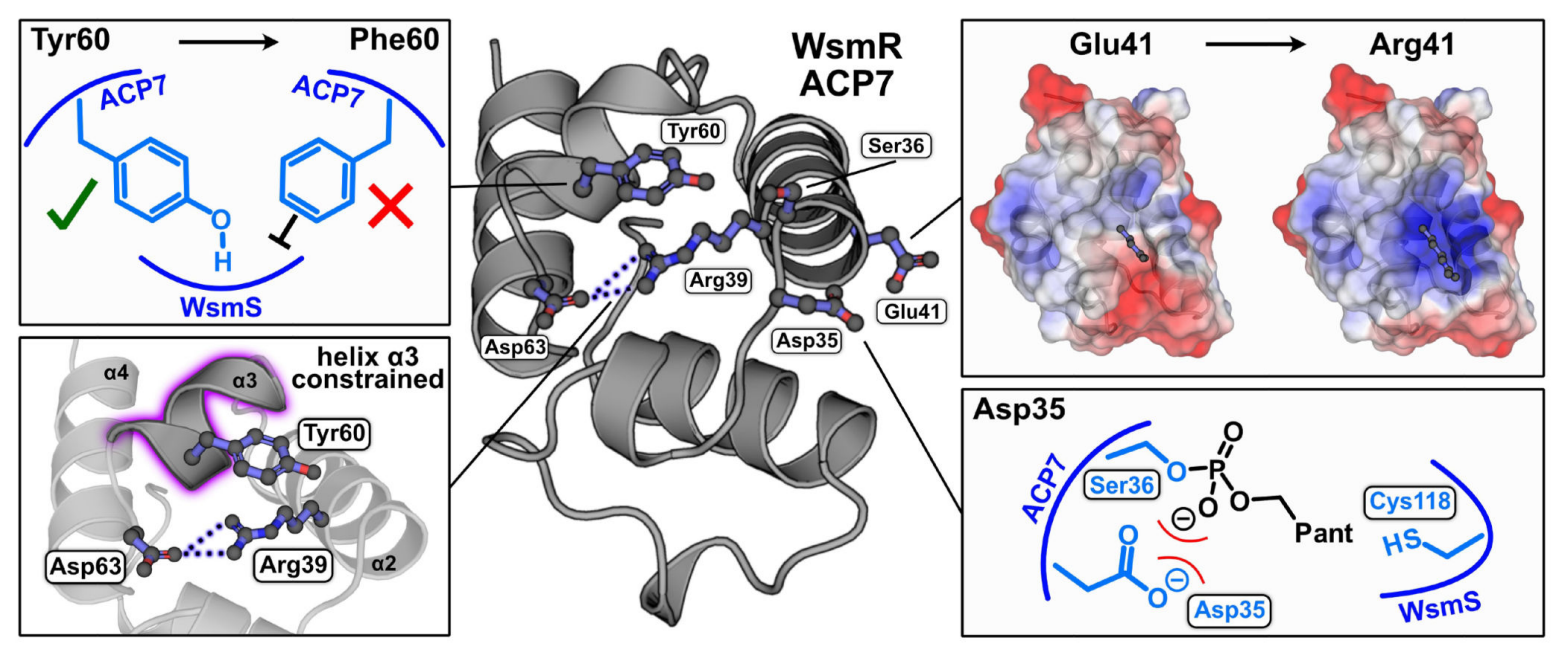
An AlphaFold2 model of WsmR ACP7 was used to visualize critical conserved module 8 ACP_A_ residues.^[25]^ Residues are numbered in accordance with the excised WsmR ACP7 domain. The importance of each residue for flagging WsmS is hypothesized or illustrated based on previous structural studies and are representative of the results shown in Figure S18.^[11,14]^

With the roles of WsmR ACP7 and ACP8 in β-branching confirmed, investigations turned to identification of molecular features that could govern their functional inequivalence. Sequence logos of leinamycin-type module 8 ACPs used for phylogenetic analysis (Figure S2) were generated based on their clade: clade 1 referring to ACP_A_s and clade 2 referring to non-ACP_A_s (Figure S16). We noted several differences in conserved residues between the two clades and the key ACP_A_ residues were visualized using an AlphaFold2 model of WsmR ACP7 (Figure 6).^[25]^ This revealed that five of the conserved residues encompass the N-terminal portion of helix α2, helix α3 and the α3-α4 loop, which constitutes the ACP_A_ docking interface for characterized ACP complexes with β-branching enzymes from curacin and virginiamycin biosynthetic pathways.^[10,14,26]^ These residues were therefore identified as potentially important sequence motifs for module 8 β-branching in leinamycin-related biosynthetic pathways. Asp35, Asp/Glu41 and Tyr60 are fully conserved in clade 1 and their importance in β-branching previously identified or speculated in the mupirocin and virginiamycin biosynthetic pathways.^[11,14]^ They are largely exchanged for Thr/Ser32, Arg/Lys38 and Phe56 in clade 2, respectively. The WsmR ACP7 AlphaFold2 model also suggested that Arg39 (100 % conserved) and Asp63 (97 % conserved) in clade 1 could form an electrostatic interaction due to their close proximity. This putative interaction could constrain helix α3, which bears the conserved Tyr60 residue. Arg39 and Asp63 are substituted by Met36 and Pro59, respectively, in non-ACP_A_s.

To explore the importance of this network of conserved residues in clade 1, six WsmR ACP7 mutants were generated by site-directed mutagenesis: Y60F, D35S, E41A, E41R, R39A and D63A. All of the WsmR ACP7 variants were soluble and confirmed to be monomeric by analytical SEC (Figure S17). Each ACP was enzymatically converted to its acetoacetyl derivative, then incubated with acetyl-WsmT and WsmS to assess ACP_A_ capability by ESMS (Figure S18). Five out of the six mutants could not substitute for wild-type WsmR ACP7 as demonstrated by absence of a mass consistent with HMG species. HMG-E41A was observed by ESMS, although its production was significantly diminished in comparison to assays with wild-type WsmR ACP7. As a single mutation of this network of residues within WsmR ACP7 is detrimental to β-branching, these results inferred the importance of all equivalent WsmR ACP8 residues that may abrogate β-branching.

We also preliminarily assessed the ACP_A_ activity of single WsmR ACP8 S32D and F56Y mutants (equivalent to WsmR ACP7 D35S and Y60F, respectively). Unsurprisingly, these mutations were not sufficient to knock-in HMG production as judged by ESMS (Figure S19). Because the network of β-branching residues for WsmR ACP7 was significantly altered by single-point mutations, reconstitution of this network for WsmR ACP8 may be required to alter its activity.

**Figure 7:**
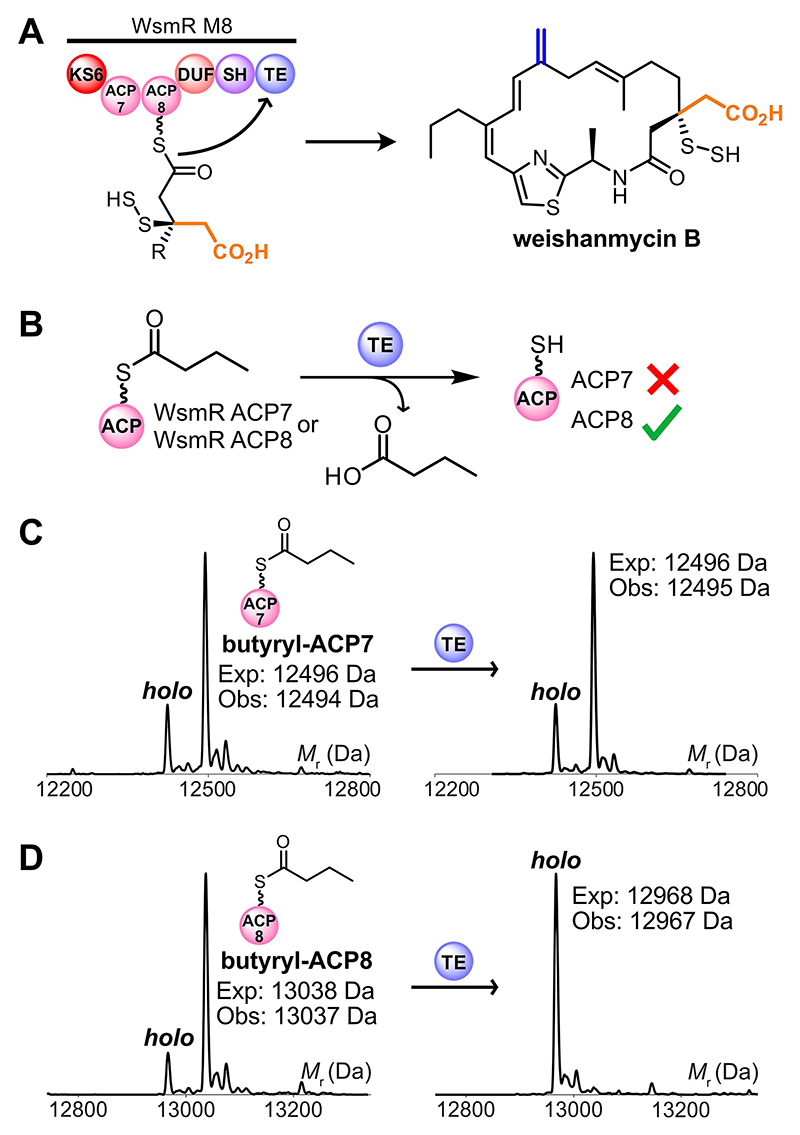
WsmR TE ESMS assays. A) Proposed reaction scheme for polyketide off-loading and cyclization in module 8. B) WsmR TE assay reaction scheme with butyryl-WsmR ACP7 or butyryl-WsmR ACP8. C) Deconvoluted spectrum of butyryl-WsmR ACP7 in the absence (left) and presence (right) of WsmR TE. D) Deconvoluted spectrum of butyryl-WsmR ACP8 in the absence (left) and presence (right) of WsmR TE.

As the role of WsmR ACP8 remained elusive, we more broadly assessed the module architecture of non-leinamycin-related PKSs that contain DUF-SH didomains.^[17]^ These PKSs are distinct from leinamycin-related systems as they do not have the same characteristic series of modules, but they utilize the same sulfur incorporation toolkit. Intriguingly, DUF-SH-containing modules from annotated non-leinamycin PKSs only harbour one ACP (Figure S20), suggesting that a single ACP is sufficient for DUF/SH catalysis. As these non-leinamycin-related modules also lack a TE, this led us to speculate that WsmR ACP8 may be required for polyketide off-loading post-sulfur incorporation. To test this, WsmR TE, which has a canonical Ser-Asp-His catalytic triad,^[27]^ was recombinantly overproduced and purified to homogeneity (Figure S20). Analytical SEC suggested that the excised TE existed as a monomeric species (Figure S20). WsmR ACP7 and ACP8 were enzymatically derivatized with butyryl-CoA and incubated with WsmR TE to assess substrate hydrolysis by ESMS (Figure 7B). We found that no TE-catalyzed hydrolysis of butyryl-WsmR ACP7 occurred based on comparison to a control assay excluding WsmR TE (Figure 7C), whereas complete hydrolysis of butyryl-WsmR ACP8 was observed in the presence of the TE (Figure 7D). Importantly, this provided further evidence of an in-series mechanism regulating biosynthesis in module 8. The TE was able to off-load several short acyl substrates from WsmR ACP8, including a malonyl group, but HMG hydrolysis was minimal (Figure S20). None of the acyl substrates tested were off-loaded from WsmR ACP7 (Figure S20), suggesting that substrate transfer from WsmR ACP7 to ACP8 must occur pre- or post-sulfur incorporation to ensure that successful chain release and cyclization occurs.

Because there were no candidate module 8 domains that could catalyze transacylation, we tested if spontaneous substrate transfer could occur between the excised ACPs prior to sulfur incorporation. To do so, HMG-WsmR ACP7 was incubated with *holo*-WsmR ACP8 in the presence of WsmE (Figure S21). We did not observe any evidence of MG transfer from WsmR ACP7 to *holo*-WsmR ACP8 by ESMS. Furthermore, additional transacylation assays with several short acyl substrates (e.g., acetoacetyl) failed to show transfer from ACP7 to ACP8 (data not shown). The lack of observable transfer could point to an alternate, unknown mechanism for transacylation.

**Figure 8:**
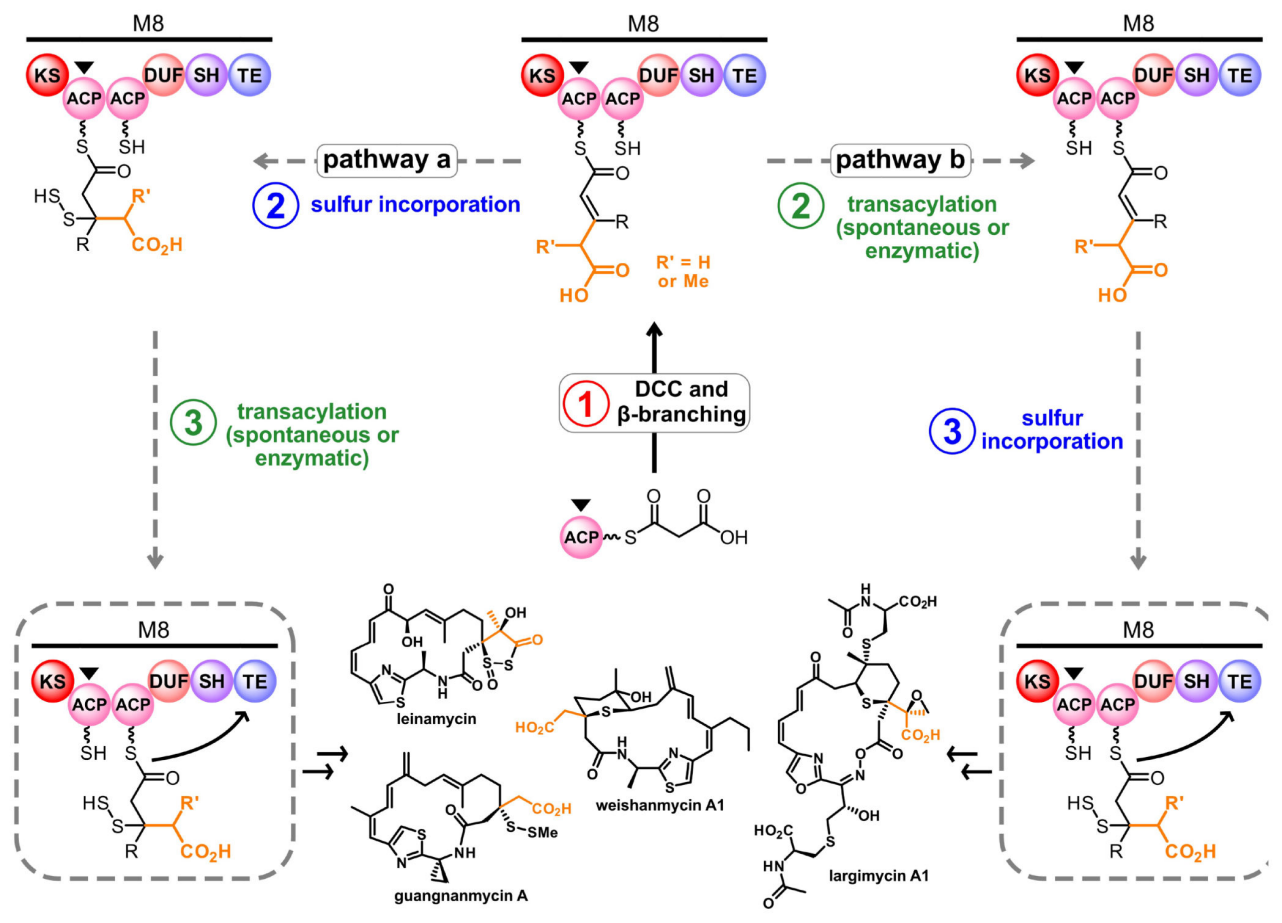
A new proposal for the final stages of leinamycin-related biosynthesis. After DCC and β-branching on the first tandem ACP, sulfur incorporation could occur before (pathway a) or after (pathway b) substrate transfer to the second tandem ACP. The final assembly line product is off-loaded from this ACP by the C-terminal TE.

Taken together, these studies support an unprecedented in-series mechanism in the final stages of assembly line production of leinamycin-type polyketides (Figure 8). After malonylation of the first tandem ACP, DCC and β-branching occurs to give a carboxylated β-branch intermediate. Sulfur incorporation could feasibly occur on the same ACP prior to polyketide chain translocation (pathway a) or on the second tandem ACP following polyketide chain translocation (pathway b) (Figure 8). Once the hydropersulfide moiety is introduced, the polyketide chain is off-loaded from the second tandem ACP and cyclized by the C-terminal TE.

The ESMS assays have directly established the divergent roles of the WsmR ACP didomain for the first time, showing that DCC and β-branching in module 8 exclusively occurs on WsmR ACP7. Furthermore, we have confirmed that WsmD is selective for WsmR ACP4, and WsmS for WsmR ACP7. The requirement for multiple HMGSs is not unique to the leinamycin family. Two HMGSs (TaC and TaF) are employed in the myxovirescin biosynthetic pathway to install two distinct β-branches via individual ACP_A_s.^[28]^ However, in vivo and in vitro studies have shown that TaC can complement for TaF.^[29]^ Conversely, our in vitro ESMS assays show that WsmD and WsmS are highly specific and cannot complement each other. Kinetic and steric control mechanisms must therefore efficiently regulate myxovirescin β-branching via alternate HMGSs.

Previous phylogenetic analysis of leinamycin-type HMGSs suggested that they clade according to the module in which the β-branch is installed.^[15]^ WsmS clades with LnmM, the HMGS responsible for β-branching in module 8 of the leinamycin biosynthetic pathway, whereas WsmD clades separately and was therefore hypothesized to install the C-9 *exo*-β-methylene branch. In vivo knockouts of the largimycin HMGSs, *lrgM1* and *lrgM2*, supported this hypothesis by validating their selectivity for LrgJ module 8 and module 5, respectively.^[18]^ However, functional inequivalence of the tandem ACPs was not proposed or demonstrated. The largimycin biosynthetic gene cluster (BGC) encodes two substrate- and HMGS-specific ACP_D_s: acetyl-LrgA cooperates with LrgM2, and propionyl-LrgL with LrgM1.^[19]^ In contrast, WsmT is the sole ACP_D_ encoded by the weishanmycin BGC. The requirement for two HMGSs is therefore necessitated by ACP_A_ specificity in the weishanmycin biosynthetic pathway, whereas a combination of ACP_D_ and ACP_A_ specificity appears to modulate β-branching in the largimycin biosynthetic pathway.

Examples and evidence for functionally inequivalent tandem ACPs in polyketide biosynthesis are only now beginning to emerge. A recent biophysical study of the (non-leinamycin-related) virginiamycin biosynthetic pathway proposed that the β-branching didomain ACPs, VirA ACP_5a/b_, are divergent.^[14,30]^ In contrast to the weishanmycin β-branching pathway, the second ACP, ACP_5b_, was shown to interact with β-branching enzymes using tryptophan fluorescence quenching assays, while ACP_5a_ showed no or very weak binding. The surface charge distribution of ACP_5b_, alongside α1-α2 loop residues Ala6862 and Asn6865, were shown to be important for recognition. Based on these biophysical assays, an in-series mechanism was proposed, in which chain extension occurs on ACP_5a_ prior to transfer of the β-ketothioester intermediate to ACP_5b_ and subsequent β-branching. Here, we definitively demonstrate that the analogous steps in the weishanmycin biosynthetic pathway follow a different path, with DCC and β-branching confined to a single ACP. To date, this mechanism is unique to the leinamycin family and highlights the exciting possibility that several contrasting mechanisms of functionally inequivalent ACP didomains may have evolved. This warrants a broader reanalysis of PKSs to study how these in-series mechanisms may have emerged.

Bioinformatic analysis and site-directed mutagenesis have uncovered five residues that distinguish the roles of the tandem ACPs in the leinamycin family (Figure 6). We identified a network of ACP_A_ residues mainly located on helices α2 and α3, and their importance in flagging WsmS was verified. While key Vir_A_ ACP_5b_ residues for β-branching enzyme interactions were largely located on the α1-α2 loop, this region showed little conservation in leinamycin-type module 8 ACP_A_s, further supporting a divergent in-series mechanism in the assembly of leinamycin polyketides.^[14]^

Previous studies have hypothesized that Tyr60 and Asp35 are critical for β-branching in other biosynthetic pathways.^[11,14]^ Tyr62 (analogous to Tyr60) in MmpA ACP_3a_, an ACP_A_ in the mupirocin biosynthetic pathway, resulted in a three-fold reduction in antibiotic production when sub-stituted for a Phe residue in vivo.^[11]^ 83 % of leinamycin-type non-ACP_A_s bear a Phe residue at this position. The equivalent Asp35 residue in VirA ACP_5b_ (Asp6870) was identified at the *holo*-ACP_5b_/VirD (ECH_1_) docking interface (PDB ID: 8AHQ) and participated in a complex hydrogen bonding network that constrained its carbonyl side chain.^[14]^ This consequently minimized electrostatic interactions with the Ppant arm which orients to form favourable interactions with the ECH_1_. Asp35 is exchanged for Ser/Thr in module 8 non-ACP_A_s which may negatively alter the positioning of the Ppant arm. Tyr60 and Asp35 are highly conserved amongst known ACP_A_s which may signify their widespread importance across many β-branching pathways (Figure S16).^[11]^ VirA ACP_5b_ Glu6876 (equivalent to Glu41) was also identified at the ACP_5b_/VirD docking interface.^[14]^ The WsmR ACP7 E41R mutant abolished HMG production, suggesting that the local electropositive region introduced by Arg41 disfavours HMGS recruitment. Glu41 therefore appears to be important for flagging HMGS cassette enzymes in the virginiamycin and leinamycin-type biosynthetic pathways. The conserved Arg/Lys residue at the equivalent position in module 8 non-ACP_A_s could hinder HMGS interactions. Finally, the significance of Arg39 and Asp63, which are absent in leinamycin-type non-ACP_A_s, was established. Although structural investigations would be required to provide further mechanistic insights, the positioning of helix α3 and the critical Tyr60 residue could be regulated by an interaction between these two residues. Intriguingly, this molecular feature appears to be unique to the leinamycin-type module 8 ACP_A_s (Figure S16). The absence of equivalent residues in module 5 ACP_A_s may also explain their differential HMGS selectivity and requires further investigation. Having demonstrated that β-branching exclusively occurs on WsmR ACP7, yet TE-mediated chain release is governed by WsmR ACP8, the mechanism of ACP7-to-ACP8 polyketide chain transfer, and whether this occurs pre- or post-sulfur incorporation, is a key unanswered question. Direct ACP-to-ACP polyketide chain transfer has been demonstrated in type II PKS systems, and the actinorhodin (act) ACP can transfer a malonyl group to other type II PKS ACPs in vitro.^[31]^ However, the act ACP Arg72 residue that was found to be important for transferase activity is not conserved in the leinamycin-type module 8 didomain ACPs. Spontaneous transacylation is also hypothesized to occur in the pacidamycin biosynthetic pathway, a uridyl peptide that is assembled by an NRPS.^[32]^ Evidence for direct transacylation between type I PKS ACPs is less prominent, although a “chain skipping” mechanism has been hypothesized in module 6 of the leinamycin PKS.^[33]^ Instead, polyketide chain transfer from an ACP to a downstream *holo*-ACP in type I PKSs is typically mediated by a gatekeeping non-elongating KS^0^ domain.^[3d,34]^ Control mechanisms must therefore exist to ensure that *holo*-ACPs are available for substrate transfer and prevent premature termination of biosynthetic pathways.^[35]^ No *trans*-acting KS^0^s are encoded by leinamycin-related BGCs except for those belonging to HMGS cassettes that catalyze decarboxylation rather than transacylation.^[15]^ ACP-to-ACP substrate transfer could therefore be catalyzed by an unknown transferase, or occur spontaneously. Although the latter was not observed between individual module 8 ACPs with the substrates tested, the native ACP didomain linker and/or authentic substrate may be required for translocation to occur. Importantly, if WsmR ACP8 is malonylated in vivo, the TE is able to remove this and therefore liberate *holo*-WsmR ACP8 for acceptance of a polyketide intermediate. Within the leinamycin family, it is plausible that the terminal TE domain encodes a dual function, catalyzing both malonyl cleavage from the second tandem ACP, and chain release post-sulfur incorporation.

## Conclusions

In summary, we have for the first time provided functional evidence of divergent tandem ACPs via in vitro reconstitution of some of the final on-line stages of weishanmycin biosynthesis. The functional inequivalence of these didomain ACPs necessitates in-series polyketide processing within module 8 of the leinamycin family of PKSs. DCC and β-branching is confined to the first tandem ACP, and we have identified a network of critical residues that govern β-branching within this ACP clade. The stringent HMGS selectivity in the weishanmycin β-branching pathways has also been verified, highlighting the multiple control mechanisms that prevent erroneous β-branching within this family. Now that the functional intricacies have been defined for the weishanmycin HMGS cassette, ongoing structural characterization and in vivo investigations will provide further mechanistic insights. Following sulfur incorporation, the polyketide intermediate must be off-loaded from the second tandem ACP by the C-terminal TE. This requires cryptic transacylation pre- or post-sulfur incorporation, and the TE may enable such a mechanism via malonyl hydrolysis. The precise timing and mechanisms of sulfur incorporation and polyketide chain transacylation are the current focus of ongoing investigations and will be essential in completely delineating the final biosynthetic steps in the assembly of leinamycin-type polyketides. The unprecedented divergence of the tandem ACPs within the leinamycin family warrants an extensive reanalysis of PKSs to explore how in-series processing may have emerged and evolved, and will provide exciting prospects for the logical reprogramming of PKSs that should be explored.

## Supplementary Material

Supporting Information

## Data Availability

The data that support the findings of this study are available from the corresponding author upon reasonable request.
