# Supplementary material for: Divergent Tandem Acyl Carrier Proteins Necessitate In-Series Polyketide Processing in the Leinamycin Family
Source: Angew Chem Int Ed Engl. Author manuscript; Available in PMC 2025 Aug 13. (PMC7618002; doi:10.1002/anie.202414165)
Supplement: Supporting Information [file EMS207813-supplement-Supporting_Information.pdf]

Angewandte Chemie
Eine Zeitschrift der Gesellschaft Deutscher Chemiker

Supporting Information

Divergent Tandem Acyl Carrier Proteins Necessitate In-Series Polyketide Processing in the Leinamycin Family

A. P. Phillips, A. J. Winter, C. M. Hooper, C. Williams, J. Crosby, C. L. Willis, M. P. Crump**

Supporting Information

Divergent Tandem Acyl Carrier Proteins Necessitate In-Series Polyketide Processing in the Leinamycin Family

Annabel P. Phillips,[a,+] Ashley J. Winter,[a,+] Chloe M. Hooper,[a] Christopher Williams,[a] John Crosby,[a] Christine L. Willis[a,*] and Matthew P. Crump[a,*]

[a] School of Chemistry, University of Bristol, Cantock's Close, Bristol, BS8 1TS, UK

[+] These authors contributed equally

1. General

Reagents were purchased from Thermo Fisher, Sigma-Aldrich or Merck Millipore. *E. coli* competent cells were purchased from New England Biolabs (T7 Express), Sigma-Aldrich (Tuner (DE3)) or Takara Bio (Stellar). All plasmids encoding the enzymes used were purchased from Thermo Fisher Scientific.

2. Plasmid generation

Genes were codon-optimised for expression in *E. coli* before being synthesized and sub-cloned into a pET151-D/TOPO plasmid bearing an N-terminal His_6-tag and a tobacco etch virus (TEV) cleavage site (ENLYFQ^G) by Thermo Fisher Scientific.

*LnmJ_ACP8-9*_pET151, mutant *wsmR_ACP7*_pET151 and mutant *wsmR_ACP8*_pET151 plasmids were generated from *lnmJ_ACP8-9*_S41A_pET151, *wsmR_ACP7*_pET151 and *wsmR_ACP8*_pET151, respectively, via site-directed mutagenesis with a Phusion Hot Start II High-Fidelity PCR Master Mix kit according to the manufacturer's protocol (**Table S1**). After incubating with 0.2 U/µL DpnI (37 °C, 3 hours) and ligation with T4 DNA ligase, mutated constructs were cloned using Stellar *E. coli* cells and mutations verified by Sanger sequencing (GENEWIZ).

3. Protein expression

Individual plasmids encoding all ACPs, WsmE, WsmR KS6, WsmR TE and LnmF were transformed into *E. coli* T7 Express cells. Cultures were grown in LB media supplemented with carbenicillin (100 µg/mL) at 37 °C with shaking until the OD_{600} = 0.6. Expression was induced with 0.25 mM isopropyl β-D-1-thiogalactopyranoside (IPTG) at 16 °C. After 16 hours, cells were harvested by centrifugation, resuspended in buffer A (50 mM Tris-HCl pH 8.0, 500 mM NaCl, 10% (v/v) glycerol, 1 mM TCEP) and stored at -20 °C. WsmD and WsmS were overproduced using the same procedure, except Tuner (DE3) cells were used.

^{15}N-labeled proteins were produced following the same procedure except cultures were grown until the OD_{600} = 2.0. Cells were pelleted by centrifugation, washed in sterile M9 media and then exchanged into fresh M9 minimal media at a 4:1 volumetric ratio. Cultures were supplemented with 0.5% (v/v) glycerol, 0.05% (w/v) glucose and 1 g/L $^{15}NH_4Cl$. Protein expression was induced with 0.25 mM IPTG at 16 °C. Cells were harvested after 16 hours, resuspended in buffer A and protein purified as described below.

LnmG, MupN and CoaA/CoaD/CoaE were overproduced and purified as described previously.[1]

4. Protein purification

Cells were lysed by sonication and the supernatant purified by Ni^{2+} immobilized metal affinity chromatography (IMAC) via a HiTrap IMAC HP 5 mL column (GE Healthcare). His_6-tagged protein was eluted using a linear gradient of 6-100% buffer B (50 mM Tris-HCl pH 8.0, 500 mM NaCl, 800 mM imidazole, 10% (v/v) glycerol) over 10 column volumes. Fractions were pooled according to SDS-PAGE analysis and further purified by size exclusion chromatography (SEC). A HiPrep 26/60 Sephacryl S-100 or S-200 column (GE Healthcare) was used in buffer C (25 mM Tris-HCl pH 7.5, 150 mM NaCl, 1 mM DTT) or buffer D (25 mM Tris-HCl pH 7.5, 500 mM NaCl, 1 mM DTT), respectively. Proteins were concentrated using centrifugal filters and either immediately used or stored at -20 °C. Analytical SEC with a Superdex 75 10/300 or Superdex 200 increase 10/300 GL column (GE Healthcare) was performed to analyse the purified protein. Molecular weight was predicted based on calibration with molecular weight standards (GE Healthcare).

His_6-WsmT was exchanged into buffer A post-IMAC and incubated with in-house TEV protease (4 °C, 16 hours) before subsequent purification by SEC to isolate cleaved protein.

5. ESMS

Samples for ESMS analysis were desalted with a 10 µL ZipTip with 0.6 µL C_4 resin (Merck Millipore) according to the manufacturer's protocol. Denatured samples were analyzed using a Synapt G2-Si (Waters) equipped with a TriVersa NanoMate® (Advion). Spectra were acquired in positive ion mode at a range of 200-3000 *m/z* using the following parameters: capillary voltage, 1.5 kV; sample cone, 10 V; trap collision energy, 10 V. Spectra were analyzed using MassLynx 4.1 software. For Ppant ejection assays, a single charge state was isolated and the trap collision energy increased to up to 20 V for sufficient fragmentation. Spectra were acquired at a range of 200-1000 *m/z*.

6. ACP derivatization and ESMS assays

CoA substrates were purchased from Sigma-Aldrich and used for derivatizing ACPs as previously described.[1b, 2] Loaded ACPs were desalted using a Zeba Spin desalt column (Thermo Fisher Scientific) prior to assays. Acetyl-pantetheine and acetoacetyl-pantetheine were synthesized as previously described.[1b, 2]

6.1. LnmG assays

80 µM *holo*-ACP (WsmR ACP7, WsmR ACP8, LnmJ ACP8 or LnmJ ACP8-9 S41A) was incubated with 5 µM LnmG and 1 mM malonyl-CoA at room temperature in buffer E (25 mM Tris-HCl pH 7.0, 250 mM NaCl, 10% glycerol, 2 mM DTT). Reactions were monitored by ESMS after 1 hour.

6.2. WsmR KS6 assays

50 μM butyryl-WsmR ACP6 was incubated with 50 μM malonyl-ACP (WsmR ACP4, ACP7 or ACP8) and 20 μM WsmR KS6 at room temperature in buffer D. Reactions were monitored by ESMS after 3 hours.

6.3. HMGS assays

50 μM acetyl-WsmT was mixed with 50 μM acetoacetyl-ACP (WsmR ACP4, ACP7 or ACP8) and incubated with 5 μM WsmD or WsmS in buffer D at room temperature. Reactions were monitored for up to 3 hours by ESMS.

6.4. ECH_1 assays

80 μM HMG-ACP (WsmR ACP4, WsmR ACP7, WsmR ACP8, LnmJ ACP8 or LnmJ ACP8-9 S41A) was incubated with 5 μM WsmE or LnmF in buffer D at room temperature. Reactions were monitored by ESMS after 1 hour.

6.5. WsmR TE assays

80 μM acyl-ACP (WsmR ACP7 or ACP8) was incubated with 5 μM WsmR TE at room temperature. Reactions were monitored by ESMS after 1 hour.

6.6. Transacylation assay

50 μM HMG-WsmR ACP7 and 50 μM *holo*-WsmR ACP8 was incubated with 5 μM WsmE at room temperature. Reactions were monitored by ESMS after 3 hours.

7. NMR parameters

^{1}H-^{15}N HSQC spectra were acquired on a Bruker AVANCE III HD 700 MHz spectrometer equipped with a 1.7 mm triple-resonance microcryoprobe. ^{15}N-labeled proteins were exchanged into NMR buffer (50 mM sodium phosphate pH 7.5, 500 mM NaCl, 1 mM DTT) and supplemented with 10% (v/v) D_2O before data acquisition at 288 K.

8. Phylogenetic analysis

Amino acid sequences of module 8 tandem ACPs from 29 leinamycin-related PKSs were manually extracted by analysing their corresponding genomes/BGCs with antiSMASH 7.0 (**Table S2**).[3] ACPs were aligned using MUSCLE[4] before a maximum-likelihood phylogenetic tree was generated using MEGA 11[5] over 500 iterations. The LG+G+I substitution model was used with gap sites included. The tree was rooted using modular erythromycin ACPs (EryAI ACP1-3) and the bootstrapped tree visualised using FigTree.

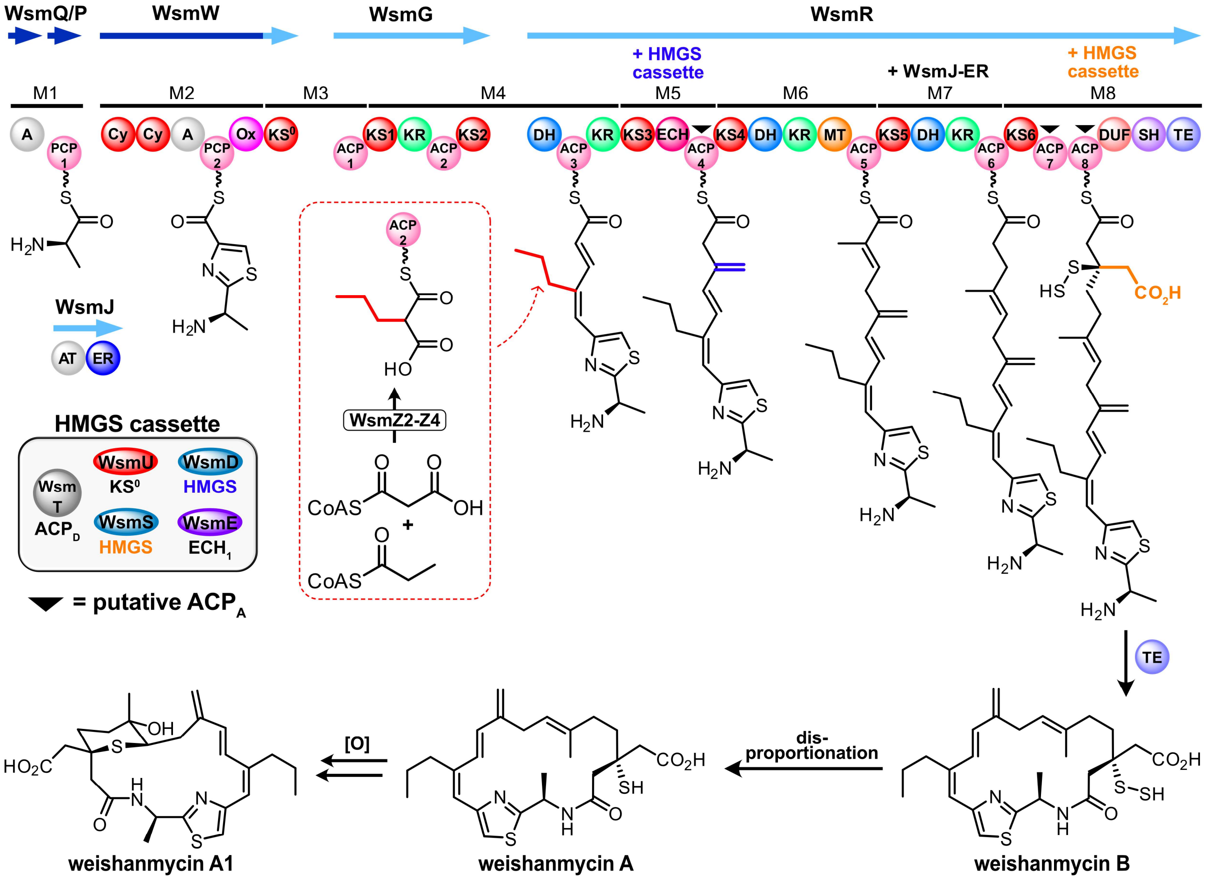

Supplementary Scheme 1. The proposed weishanmycin biosynthetic pathway.[6] Both acyl carrier proteins (ACPs) in module 8 were tentatively assigned as acceptor ACPs (ACP_As). ACPs and elongating ketosynthases (KSs) are numbered sequentially according to the assembly line. WsmZ2 (crotonoyl-CoA carboxylase/reductase), WsmZ3 (ketoacyl-ACP synthase III) and WsmZ4 (3-hydroxyacyl-CoA dehydrogenase) are implicated in the incorporation of a propyl branch in module 4. A: adenylation domain; ACP_D: donor ACP; AT: acyltransferase; Cy: cyclisation domain; DH: dehydratase; DUF: domain of unknown function; ECH: enoyl-CoA hydratase; ER: enoylreductase; HMGS: 3-hydroxy-3-methylglutaryl synthase; KR: ketoreductase; KS^0: non-elongating KS; MT: methyltransferase; Ox: oxidoreductase; PCP: peptidyl carrier protein; SH: thiocysteine lyase; TE: thioesterase.

A

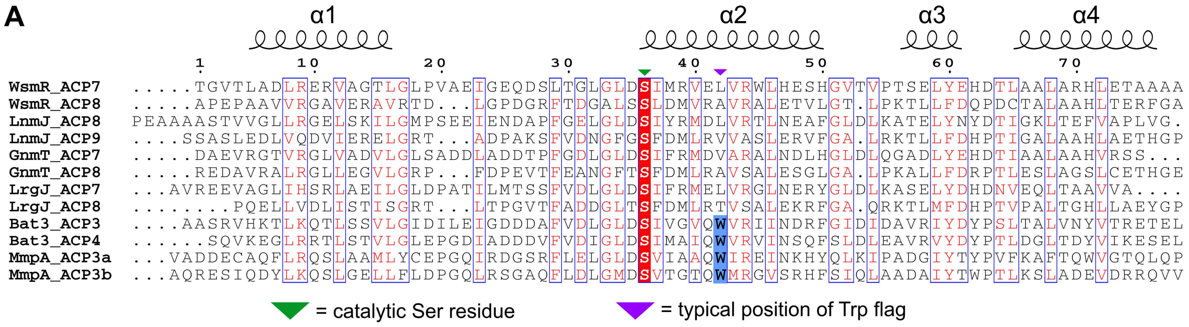

B

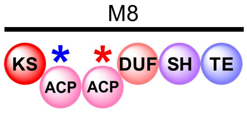

**Tandem ACPs**	MmpA ACP3a<br>MmpA ACP3b	Bat3 ACP3<br>Bat3 ACP4	WsmR ACP7<br>WsmR ACP8	LnmJ ACP8<br>LnmJ ACP9	GnmT ACP7<br>GnmT ACP8	LrgJ ACP7<br>LrgJ ACP8
**Identity/ similarity (%)**	37/60	55/83	27/46	23/41	26/42	20/38

Supplementary Figure 1. Bioinformatic analysis of leinamycin-related module 8 tandem ACPs. A) Multiple sequence alignment of WsmR, LnmJ, GnmT and LrgJ ACP didomains with representative ACP_A didomains from Bat3 (kalimantacin) and MmpA (mupirocin). The catalytic Ser residue is highlighted (green arrow) as well as the typical location of the canonical Trp flag for ACP_As (purple arrow). The Trp flag in the Bat3 and MmpA ACP_A didomains are highlighted in blue, whilst the leinamycin-related tandem ACPs lack this motif. Numbering of residues is in accordance with WsmR ACP7. Numbering of conserved WsmR and LnmJ ACP Ser residues in accordance with their PKSs are as follows: WsmR ACP7 Ser6200; WsmR ACP8 Ser6298; LnmJ ACP8 Ser6088; LnmJ ACP9 Ser6181. **B)** The sequence identity and similarity of tandem ACPs in the WsmR, LnmJ, GnmT, and LrgJ PKSs in comparison to Bat3 and MmpA ACP_A didomains. Leinamycin-related ACPs are colour coded according to their location in module 8.

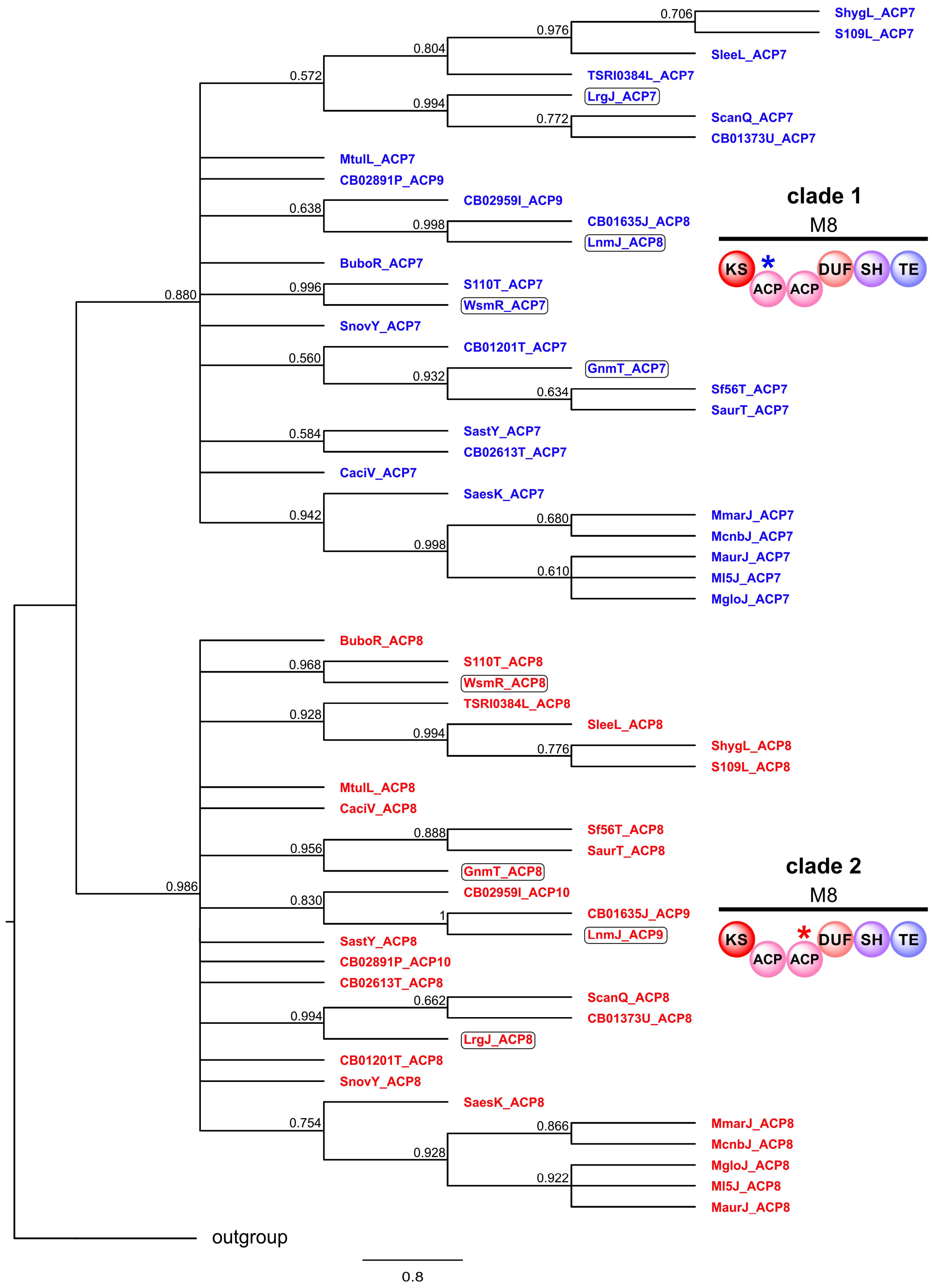

Supplementary Figure 2. Maximum likelihood phylogenetic tree of leinamycin-type ACPs from module 8 didomains. 58 ACP amino acid sequences from 29 leinamycin-type PKSs were selected to minimise clading ambiguity.[7] Three modular ACP domains from the erythromycin PKS (EryAI_ACP1-3) were used as the outgroup. Bootstrap values are shown at each node. The first tandem ACPs are shown in blue and the second tandem ACPs are shown in red. Leinamycin-type ACPs analyzed in Supplementary Figure 1 are outlined. The naming of PKSs is in accordance with studies by Pan and Becerrill.[6a, 8]

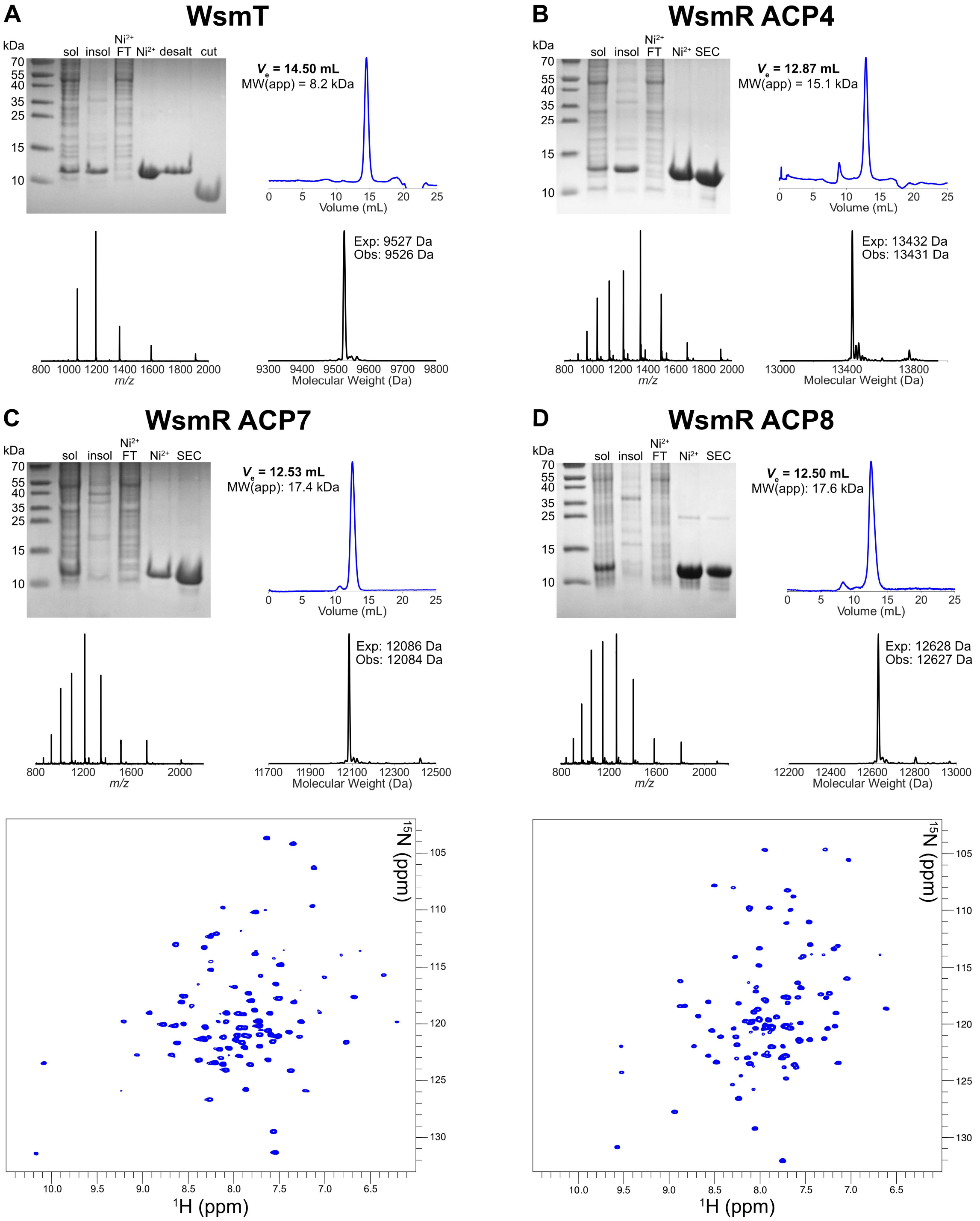

Supplementary Figure 3. Purification and characterization of WsmT and putative wsm ACP_As used within this study. A) Purification of WsmT. SDS-PAGE after purification by IMAC, followed by desalt and His_6-tag cleavage (sol: supernatant post-sonication; insol: cell pellet post-sonication; Ni^{2+} FT: IMAC flow-through; Ni^{2+}: IMAC). Analytical SEC showing elution of cleaved WsmT as a monomeric species. ESMS of denatured cleaved WsmT. **B)** Purification of WsmR ACP4. SDS-PAGE following purification by IMAC and SEC. Analytical SEC showing elution of WsmR ACP4 as a monomeric species. ESMS of denatured WsmR ACP4. **C)** Purification of WsmR ACP7. SDS-PAGE following purification by IMAC and SEC. Analytical SEC showing elution of WsmR ACP7 as a monomeric species. ESMS of denatured WsmR ACP7. ^{1}H-^{15}N heteronuclear single quantum coherence (HSQC) spectrum of ^{15}N-labeled *apo*-WsmR ACP7. **D)** Purification of WsmR ACP8. SDS-PAGE after purification by IMAC and SEC. Analytical SEC showing elution of WsmR ACP8 as a monomeric species. ESMS of denatured WsmR ACP8. ^{1}H-^{15}N HSQC spectrum of ^{15}N-labeled *apo*-WsmR ACP8.

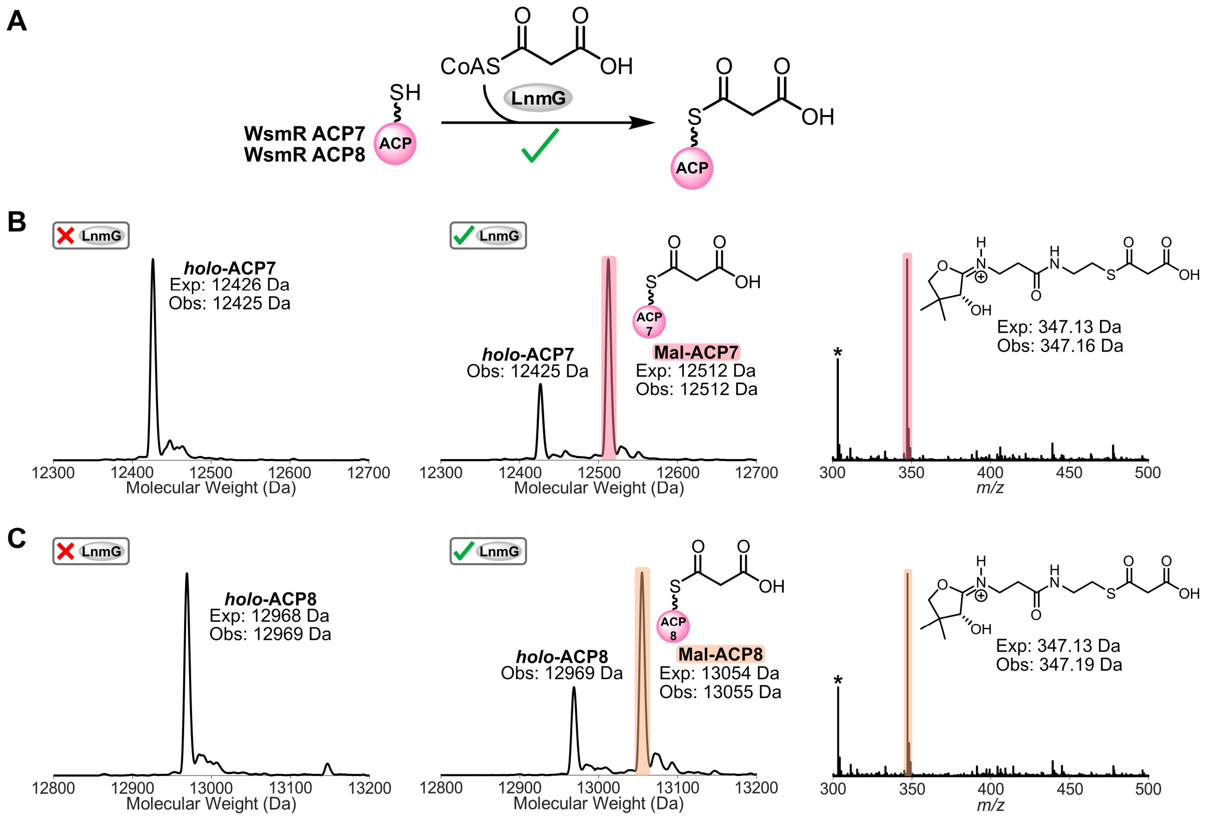

Supplementary Figure 4. LnmG malonylation ESMS assays with WsmR module 8 ACPs. A) Reaction scheme for malonylation by LnmG. **B)** Malonylation assay with *holo*-WsmR ACP7 and malonyl-CoA in the absence (left) and presence (middle) of LnmG. Ppant ejection of malonyl-WsmR ACP7 product (right). **C)** Malonylation assay with *holo*-WsmR ACP8 and malonyl-CoA in the absence (left) and presence (middle) of LnmG. Ppant ejection of malonyl-WsmR ACP8 product (right). * = acetyl species.

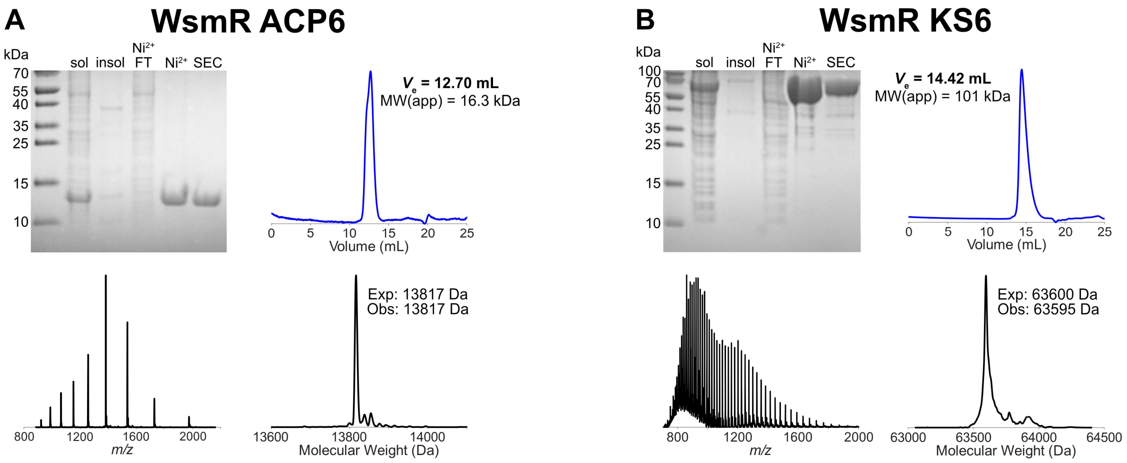

Supplementary Figure 5. Purification and characterization of WsmR ACP6 and WsmR KS6. A) Purification of WsmR ACP6. SDS-PAGE following purification by IMAC and SEC. Analytical SEC showing elution of WsmR ACP6 as a monomeric species. ESMS of denatured WsmR ACP6. **B)** Purification of WsmR KS6. SDS-PAGE following purification by IMAC and SEC. Analytical SEC showing elution of WsmR KS6 as a dimeric species. ESMS of denatured WsmR KS6.

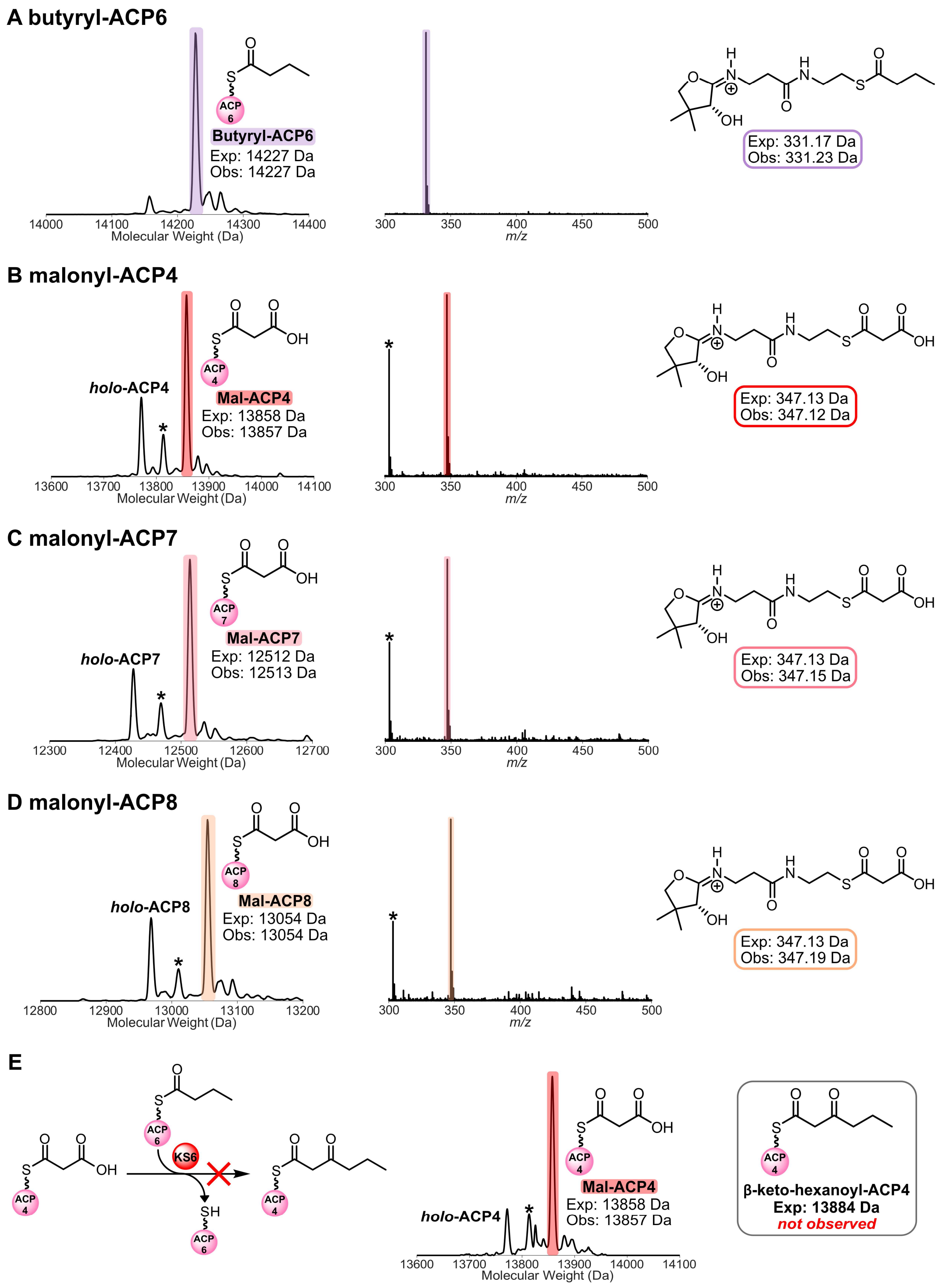

Supplementary Figure 6. Generation of wsm ACP-loaded substrates for WsmR KS6 ESMS assays. A) Deconvoluted MS (left) and Ppant ejection (middle) of butyryl-WsmR ACP6. **B)** Deconvoluted MS (left) and Ppant ejection (middle) of malonyl-WsmR ACP4. **C)** Deconvoluted MS (left) and Ppant ejection (middle) of malonyl-WsmR ACP7. **D)** Deconvoluted MS (left) and Ppant ejection (middle) of malonyl-WsmR ACP8. **E)** WsmR KS6 assay with malonyl-WsmR ACP4 as a control experiment failed to produce β-keto-hexanoyl-WsmR ACP4 (exp: 13884 Da). * = acetyl species.

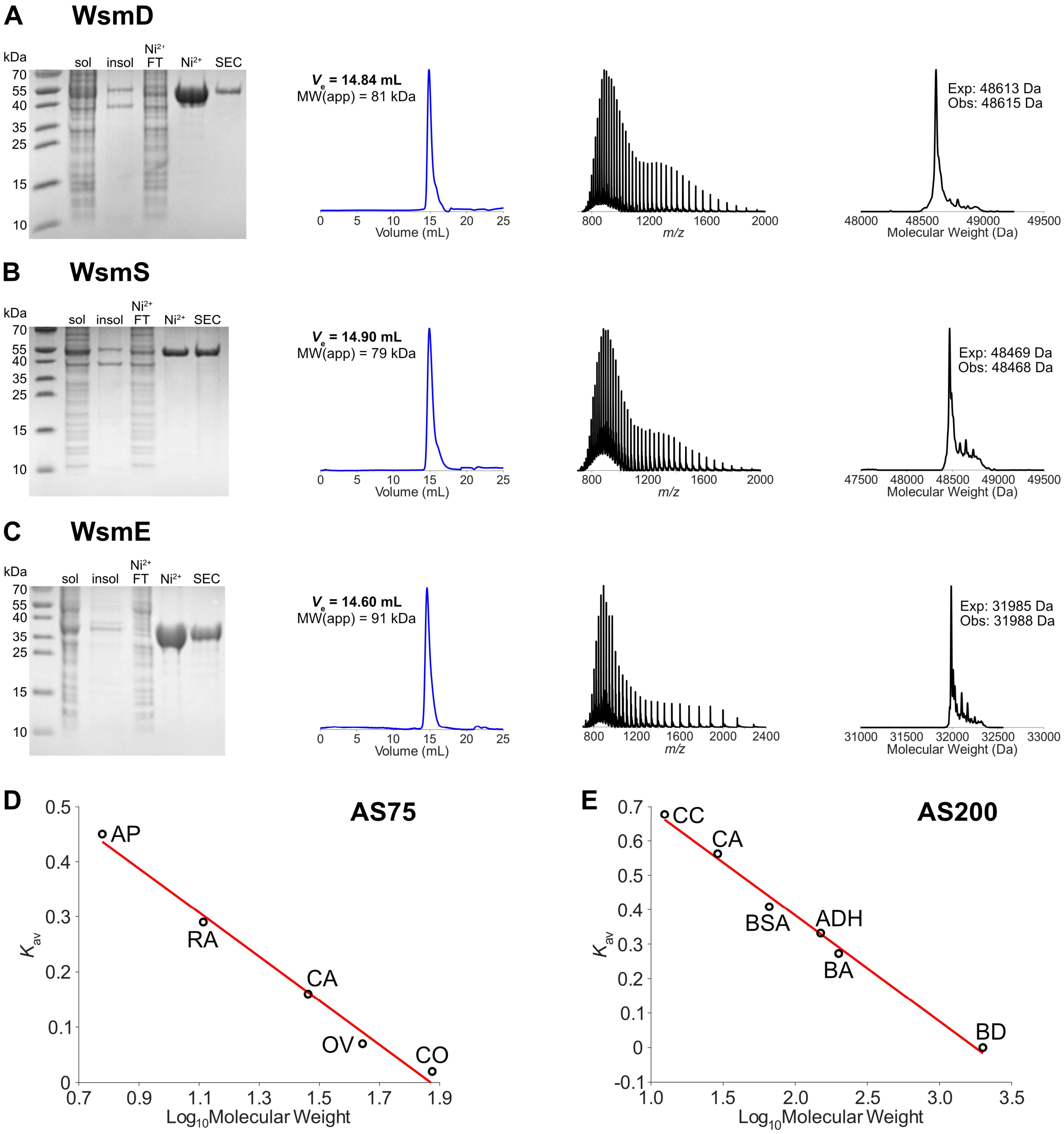

Supplementary Figure 7. Purification and characterization of all *trans*-acting wsm enzymes used within this study. A) Purification of WsmD. SDS-PAGE following purification by IMAC and SEC. Analytical SEC showing elution of WsmD as a dimeric species. ESMS of denatured WsmD. **B)** Purification of WsmS. SDS-PAGE following purification by IMAC and SEC. Analytical SEC showing elution of WsmS as a dimeric species. ESMS of denatured WsmS. **C)** Purification of WsmE. SDS-PAGE following purification by IMAC and SEC. Analytical SEC showing elution of WsmE as a trimeric species. ESMS of denatured WsmE. **D)** Calibration curve for the analytical S75 column (AP: aprotinin; RA: ribonuclease A; CA: carbonic anhydrase; OV: ovalbumin; CO: conalbumin). **E)** Calibration curve for the analytical S200 column (CC: cytochrome C; BSA: bovine serum albumin; ADH: alcohol dehydrogenase; BA: β-amylase; BD: blue dextran).

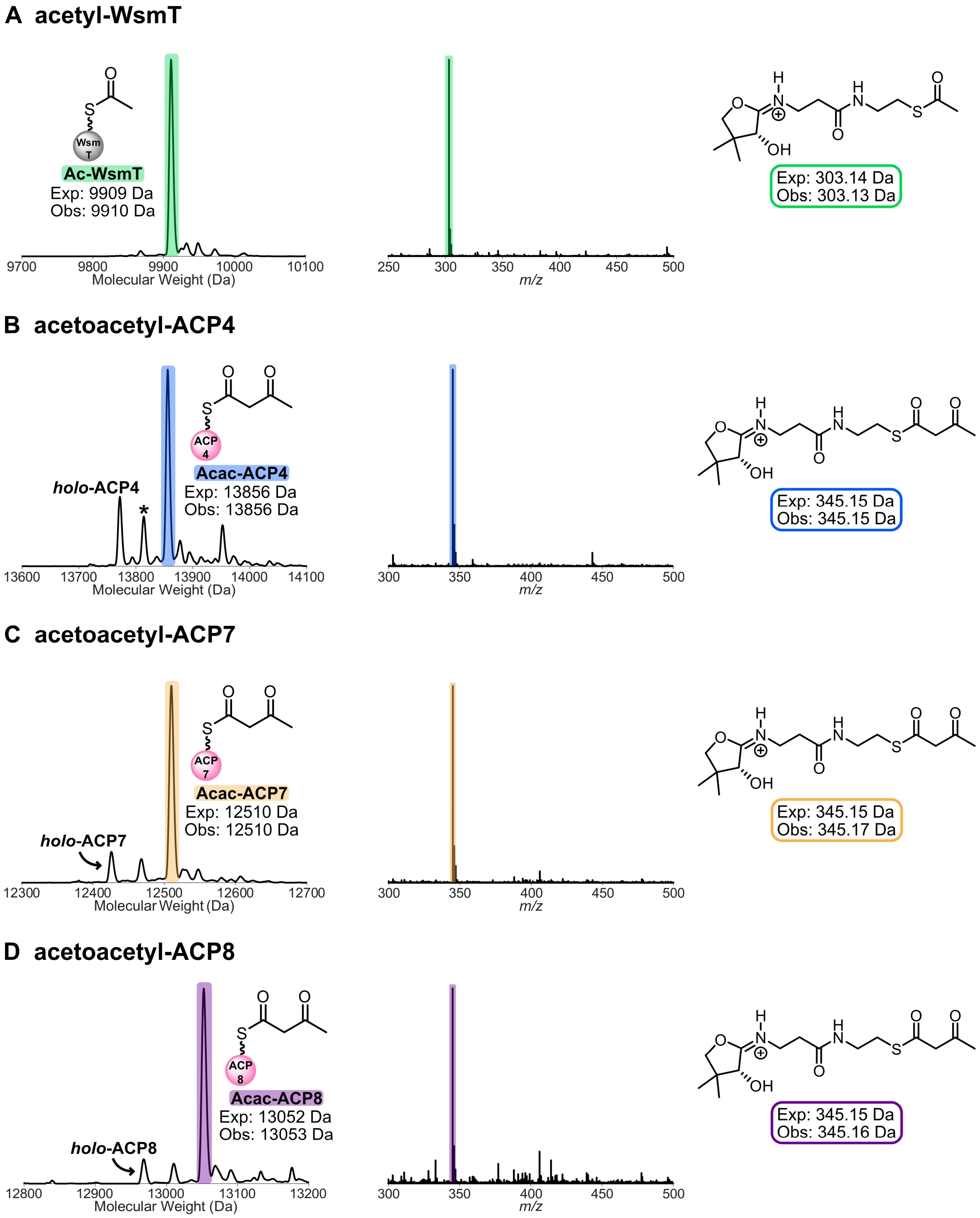

Supplementary Figure 8. Generation of wsm ACP-loaded substrates for HMGS assays. A) Deconvoluted MS (left) and Ppant ejection (middle) of acetyl-WsmT. **B)** Deconvoluted MS (left) and Ppant ejection (middle) of acetoacetyl-WsmR ACP4. * = acetyl species **C)** Deconvoluted MS (left) and Ppant ejection (middle) of acetoacetyl-WsmR ACP7. **D)** Deconvoluted MS (left) and Ppant ejection (middle) of acetoacetyl-WsmR ACP8.

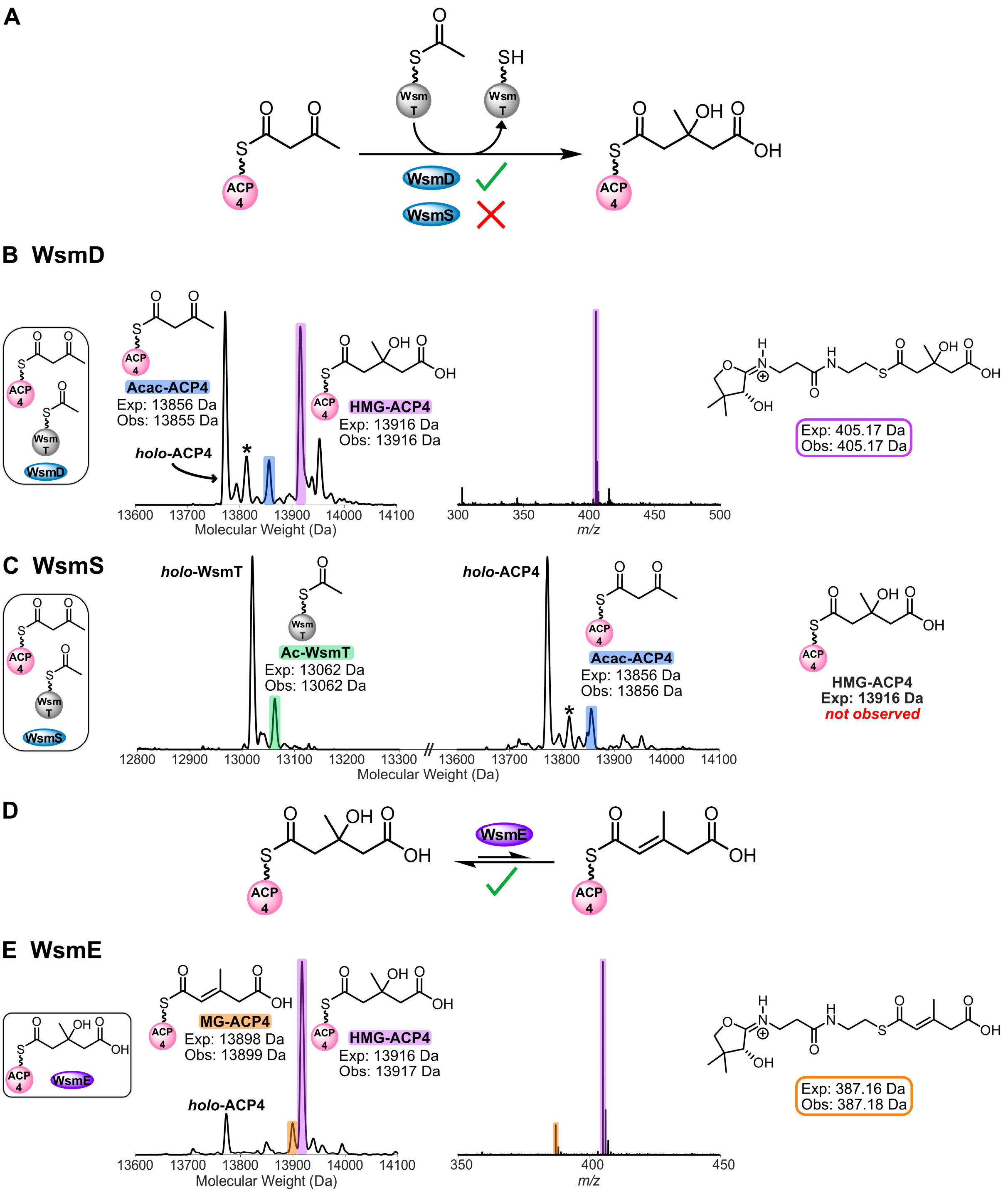

Supplementary Figure 9. β-Branching ESMS assays with WsmR ACP4. A) Reaction scheme for aldol addition of acetyl-WsmT with acetoacetyl-WsmR ACP4 catalyzed by WsmD or WsmS. **B)** WsmD ESMS assay with acetoacetyl-WsmR ACP4 yielded a new signal with a mass corresponding to HMG-WsmR ACP4 (exp: 13916 Da, obs: 13916 Da) which was verified by Ppant ejection (exp: 405.17 Da, obs: 405.17 Da). * = acetyl species. **C)** WsmS ESMS assay with acetoacetyl-WsmR ACP4. A species corresponding to HMG-WsmR ACP4 was not observed (exp: 13916 Da). **D)** Proposed reaction scheme for dehydration of (*R*,*S*)-HMG-WsmR ACP4 catalyzed by WsmE to produce the transient MG-WsmR ACP4 species. **E)** WsmE ESMS assay with HMG-WsmR ACP4 yielded a new signal with a mass consistent with MG-WsmR ACP4 (exp: 13898 Da, obs: 13899 Da). This was verified by Ppant ejection (exp: 387.16 Da, obs: 387.18 Da).

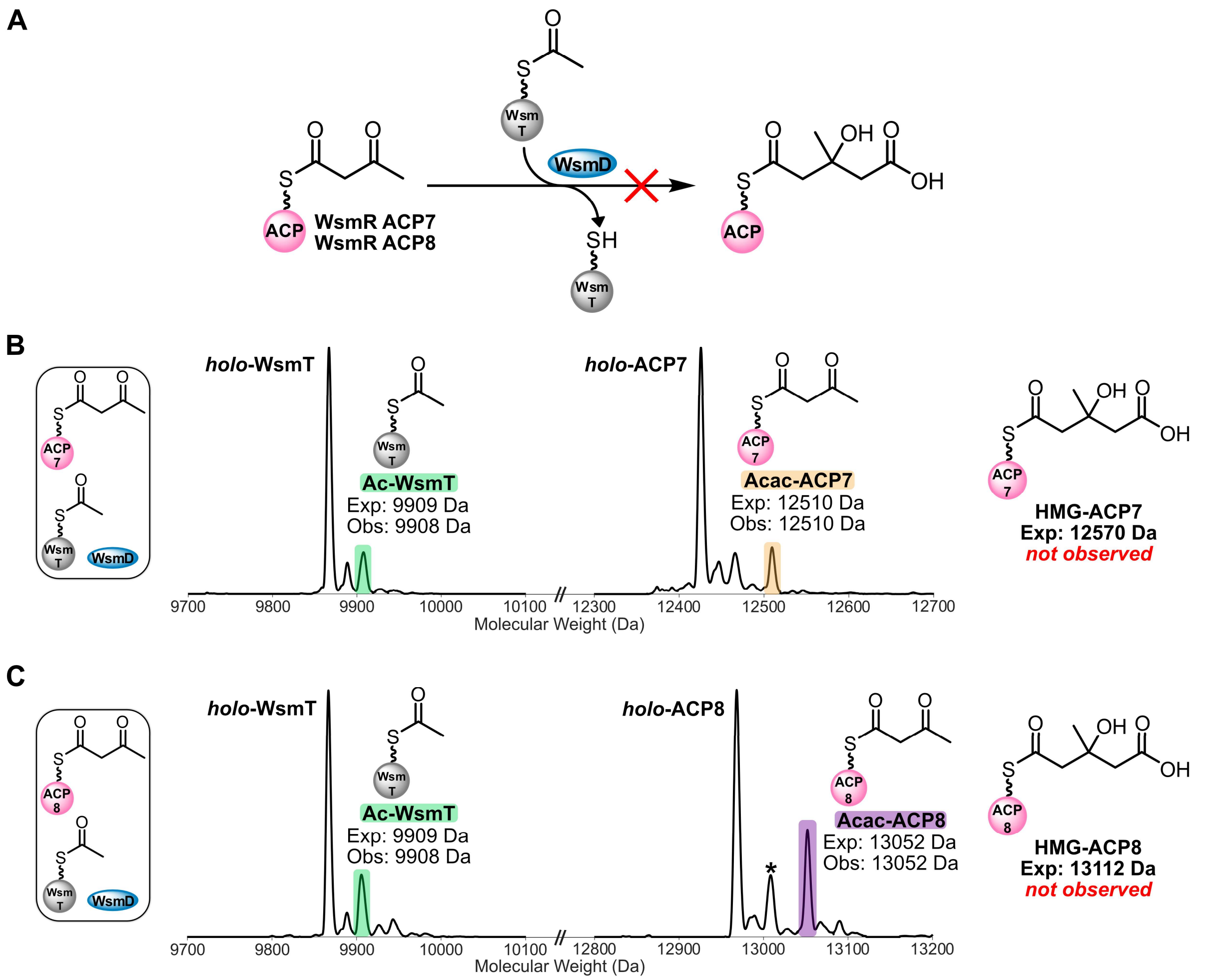

Supplementary Figure 10. WsmD ESMS assays with WsmR ACP7 and WsmR ACP8. A) Reaction scheme for WsmD-catalyzed aldol addition of acetyl-WsmT with acetoacetyl-WsmR ACP7 or acetoacetyl-WsmR ACP8. **B)** Deconvoluted MS of WsmD assay with acetoacetyl-WsmR ACP7. **C)** Deconvoluted MS of WsmD assay with acetoacetyl-WsmR ACP8. A mass corresponding to HMG-ACP was not observed for either assay. * = acetyl-ACP.

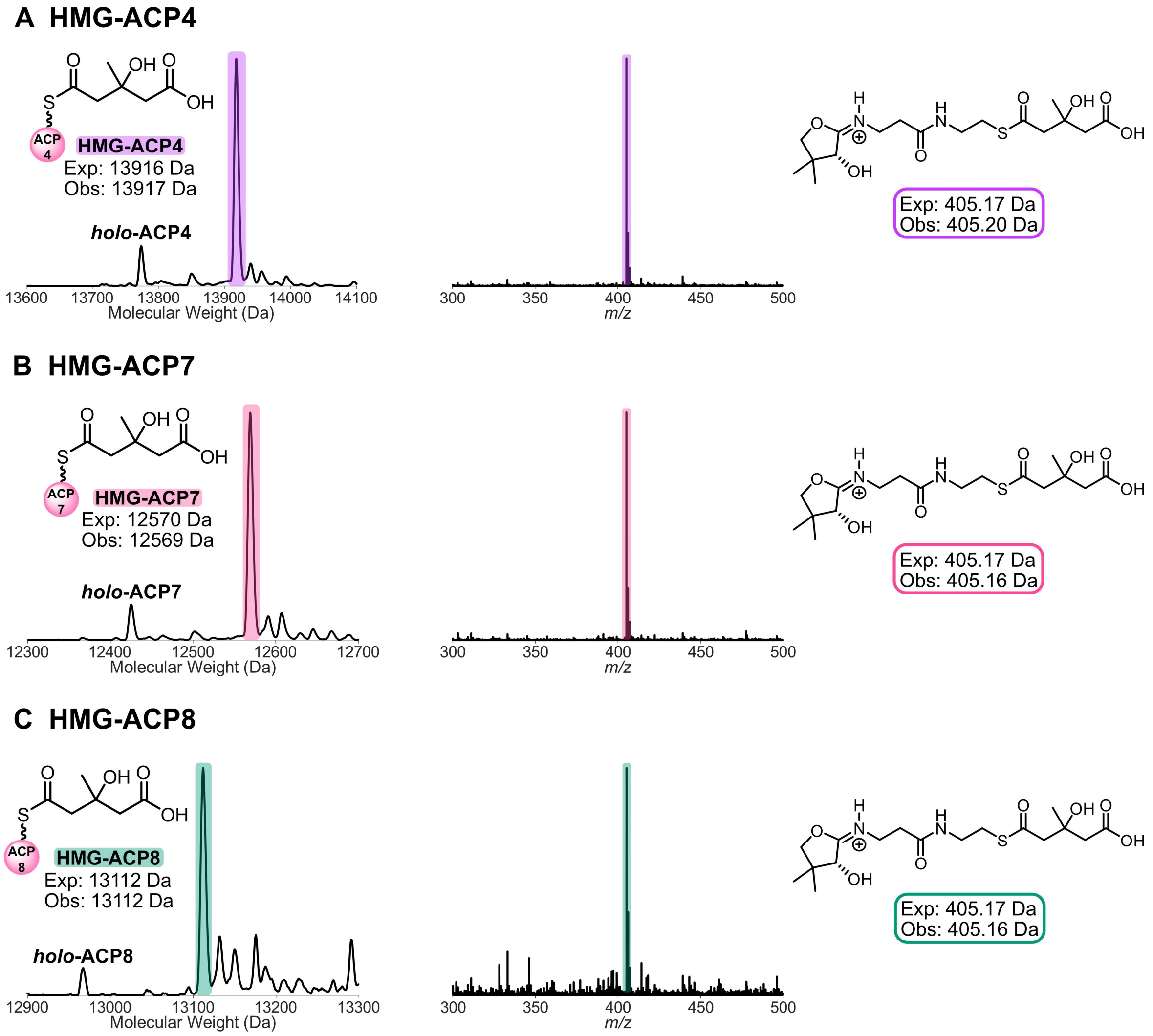

Supplementary Figure 11. Generation of wsm ACP-loaded substrates for WsmE assays. A) Deconvoluted MS (left) and Ppant ejection (middle) of (*R*,*S*)-HMG-WsmR ACP4. **B)** Deconvoluted MS (left) and Ppant ejection (middle) of (*R*,*S*)-HMG-WsmR ACP7. **C)** Deconvoluted MS (left) and Ppant ejection (middle) of (*R*,*S*)-HMG-WsmR ACP8.

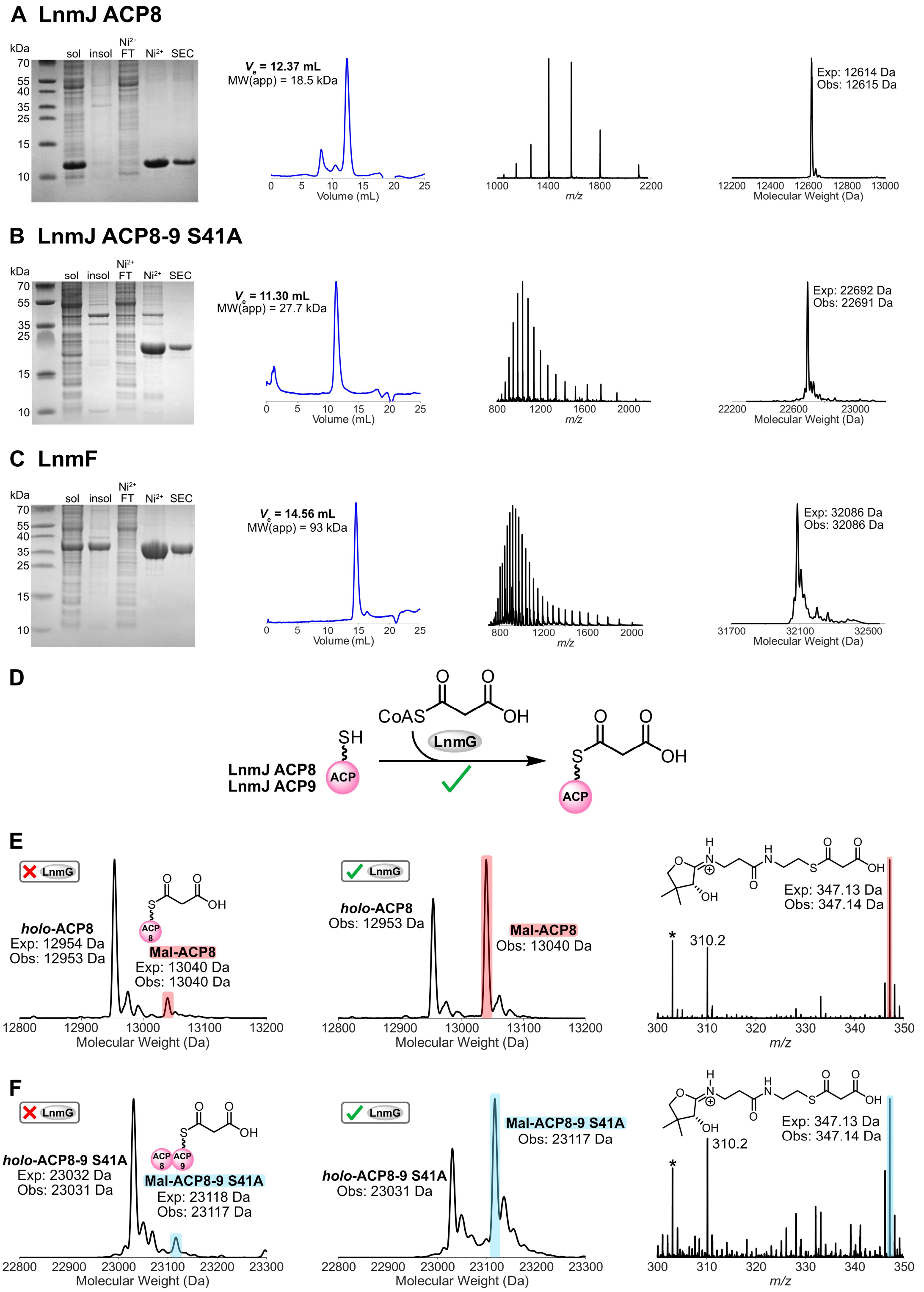

Supplementary Figure 12. Purification and characterization of lnm enzymes and LnmG malonylation assays. A) Purification of LnmJ ACP8. SDS-PAGE following purification by IMAC and SEC. Analytical SEC showing elution of LnmJ ACP8 as a monomeric species. ESMS of denatured LnmJ ACP8. **B)** Purification of LnmJ ACP8-9 S41A. SDS-PAGE following purification by IMAC and SEC. Analytical SEC showing elution of LnmJ ACP8-9 S41A as a monomeric species. ESMS of denatured LnmJ ACP8-9 S41A. **C)** Purification of LnmF. SDS-PAGE following purification by IMAC and SEC. Analytical SEC showing elution of LnmF as a trimeric species. ESMS of denatured LnmF. **D)** Reaction scheme for malonylation by LnmG. **E)** Malonylation assay with *holo*-LnmJ ACP8 and malonyl-CoA in the absence (left) and presence (middle) of LnmG. Ppant ejection of malonyl-LnmJ ACP8 product (right). **F)** Malonylation assay with *holo*-LnmJ ACP8-9 S41A and malonyl-CoA in the absence (left) and presence (middle) of LnmG. Ppant ejection of malonyl-LnmJ ACP8-9 S41A product (right). * = acetyl species.

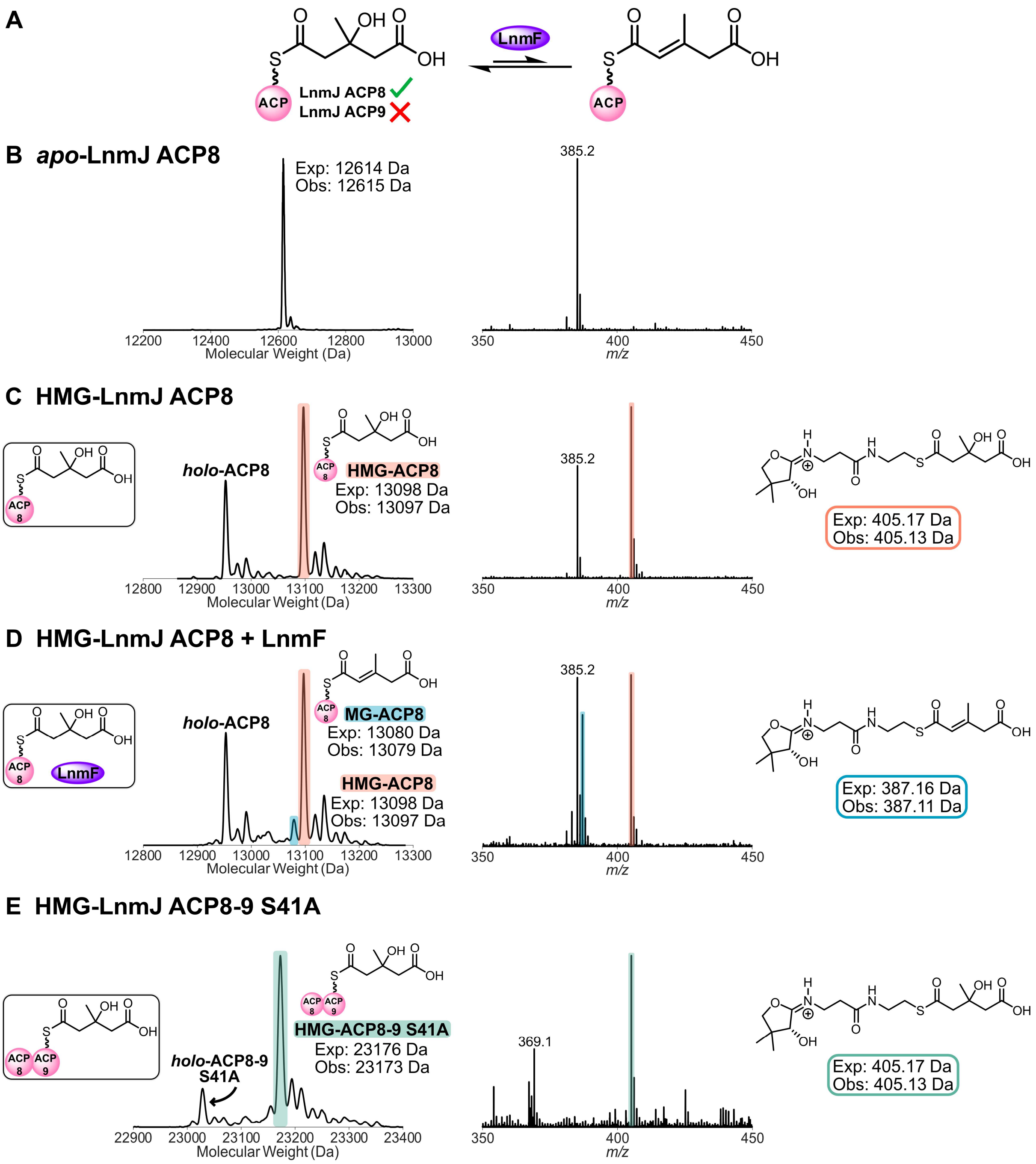

Supplementary Figure 13. Generation of lnm-loaded ACPs for LnmF ESMS assays. A) Reaction scheme for dehydration of HMG-LnmJ ACP8 or HMG-LnmJ ACP9 catalyzed by LnmF. **B)** Deconvoluted MS (left) and Ppant ejection (middle) of *apo*-LnmJ ACP8. **C)** Deconvoluted MS (left) and Ppant ejection (middle) of HMG-LnmJ ACP8. **D)** Deconvoluted MS of LnmF assay with HMG-LnmJ ACP8 (left) yielded a new mass consistent with MG-LnmJ ACP8 (exp: 13080 Da, obs: 13079 Da) which was verified by Ppant ejection (middle) (exp: 387.16 Da, obs: 387.11 Da). Additional fragmentation at 385.20 Da was observed in all samples of LnmJ ACP8. **E)** Deconvoluted MS (left) and Ppant ejection (middle) of HMG-LnmJ ACP8-9 S41A. Additional fragmentation at 369.1 Da was observed but not characterized.

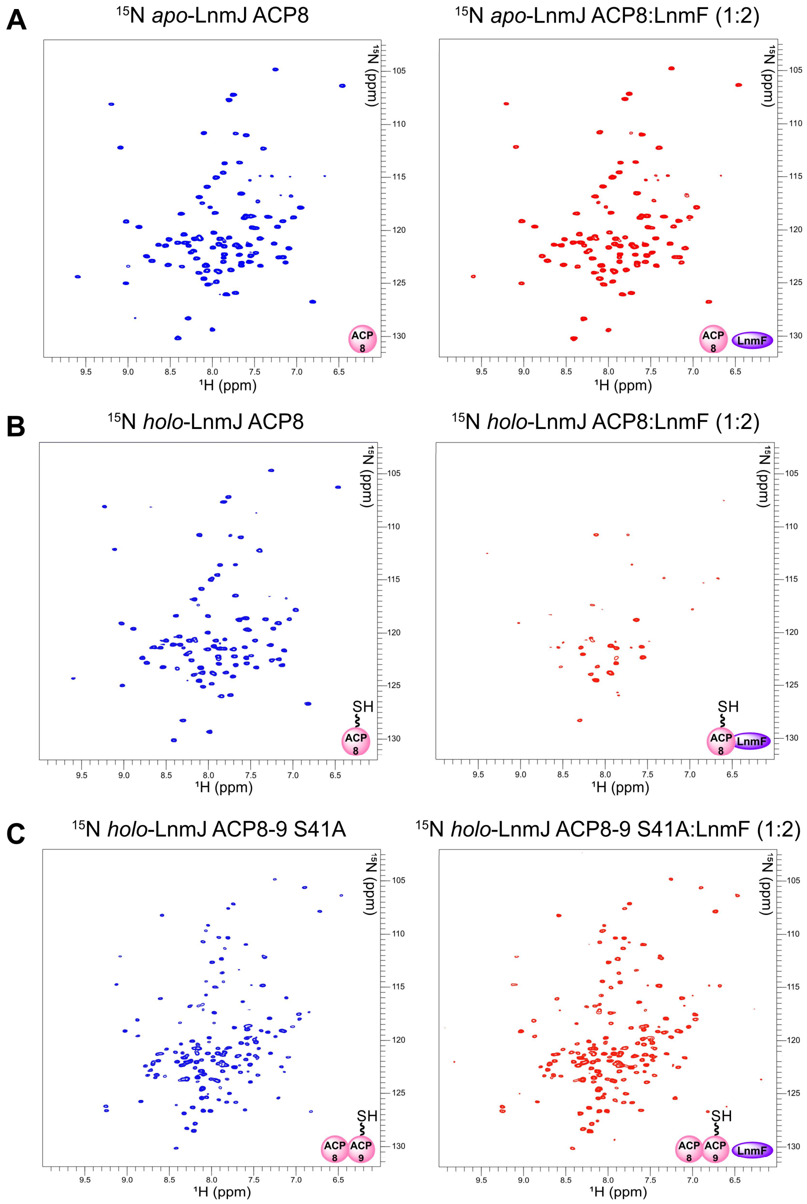

Supplementary Figure 14. LnmF NMR titrations. A) Control ^{1}H-^{15}N HSQC of ^{15}N-labeled *apo*-LnmJ ACP8 (left) and in the presence of LnmF in two-fold excess (right). No considerable resonance broadening or chemical shift perturbations (CSPs) were observed. **B)** Control ^{1}H-^{15}N HSQC of ^{15}N-labeled *holo*-LnmJ ACP8 (left) and in the presence of LnmF in two-fold excess (right). Significant resonance broadening was indicative of a protein-protein interaction. **C)** Control ^{1}H-^{15}N HSQC of ^{15}N-labeled *holo*-LnmJ ACP8-9 S41A (left) and in the presence of LnmF in two-fold excess (right). There were no discernible CSPs or resonance broadening.

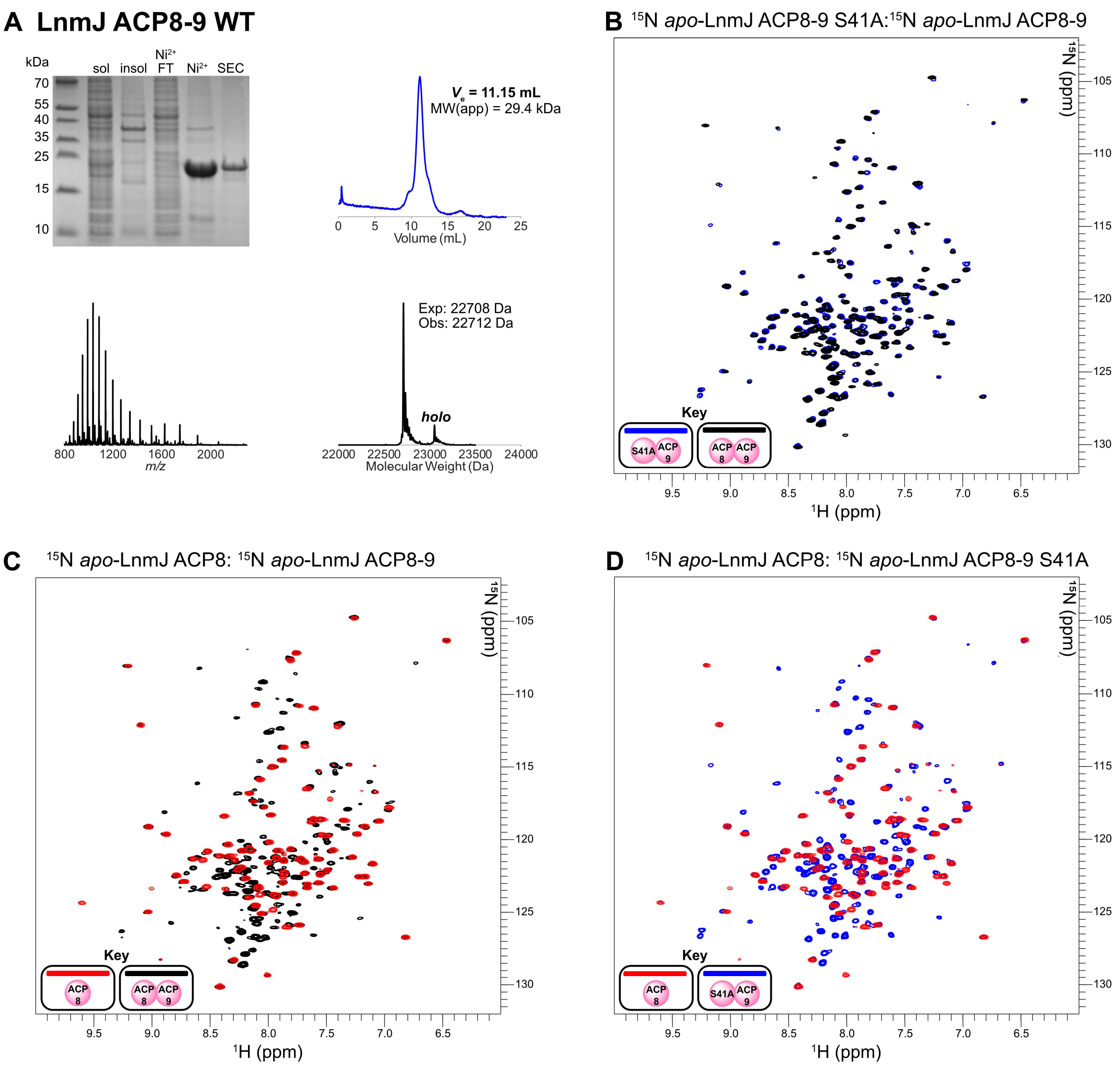

Supplementary Figure 15. Purification and characterisation of wild-type (WT) LnmJ ACP8-9 and lnm ACP NMR studies. A) Purification of WT LnmJ ACP8-9. SDS-PAGE following purification by IMAC and SEC. Analytical SEC showing elution of WT LnmJ ACP8-9 as a monomeric species. ESMS of denatured WT LnmJ ACP8-9. **B)** ^{1}H-^{15}N HSQC overlays of *apo*-LnmJ ACP8-9 S41A (black) and *apo*-LnmJ ACP8-9 (blue). Negligible CSPs suggested that the two ACP didomains were structurally similar. **C)** ^{1}H-^{15}N HSQC overlays of *apo*-LnmJ ACP8 (red) and *apo*-LnmJ ACP8-9 (black). **D)** ^{1}H-^{15}N HSQC overlays of *apo*-LnmJ ACP8 (red) and *apo*-LnmJ ACP8-9 S41A (blue). Spectra from 15C-D suggest that LnmJ ACP8 maintains its structural integrity as an excised and tandem ACP.

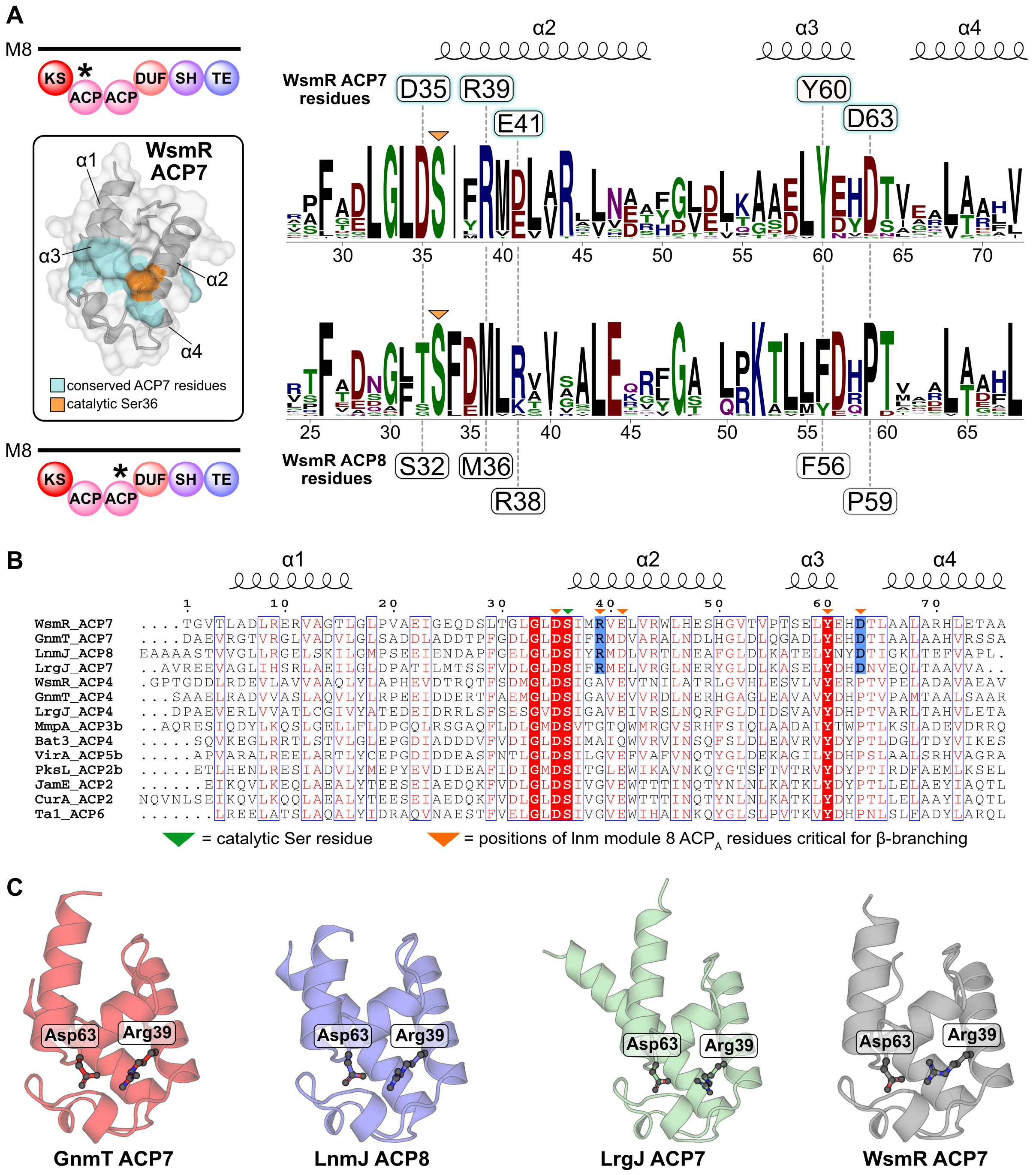

Supplementary Figure 16. A) Sequence logos of leinamycin-type module 8 didomain ACPs according to their clade. The key differences in conserved residues are annotated. A surface model of WsmR ACP7 is also shown with the corresponding conserved residues highlighted. Naming of residues is in accordance with individual WsmR ACP7 and WsmR ACP8 domains. Numbering in accordance with the WsmR PKS is as follows: D35 = D6199; R39 = R6203; E41 = E6205; Y60 = Y6224; D63 = D6227; S32 = S6297; M36 = M6301; R38 = R6303; F56 = F6321; P59 = P6324. **B)** Multiple sequence alignment of leinamycin-type module 5 and module 8 ACP$_A$s with ACP$_A$s from characterized β-branching pathways, which are named according to previous publications. The Arg39 and Asp63 residues (blue) may be unique to the leinamycin module 8 ACP$_A$s. Residue numbering is in accordance with WsmR ACP7. **C)** AlphaFold2 models of GnmT ACP7, LnmJ ACP8, LrgJ ACP7 and WsmR ACP7 showing the conserved Arg39 and Asp63 residues.[9]

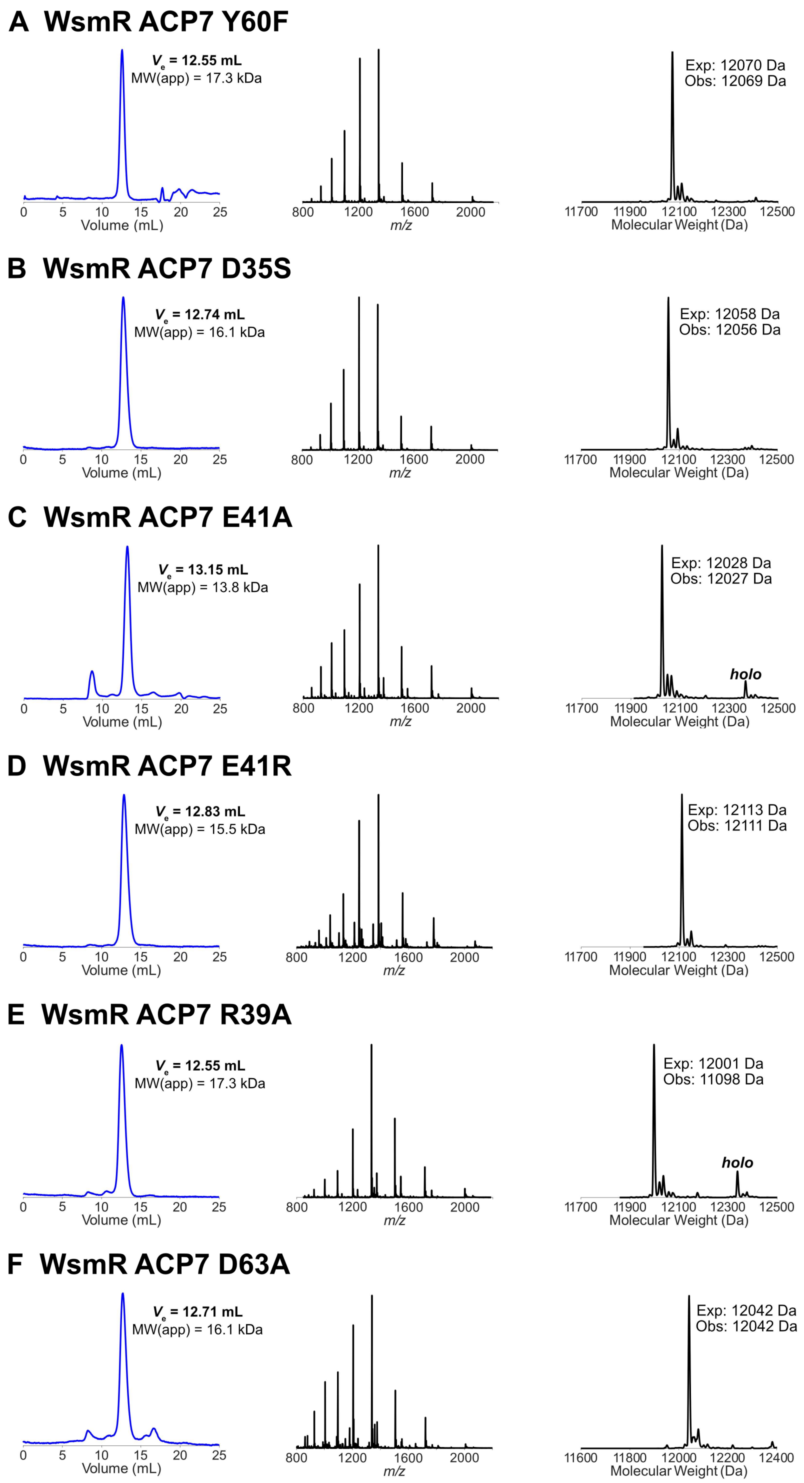

Supplementary Figure 17. Purification and characterization of all WsmR ACP7 mutants used within this study. Analytical SEC showing elution of all ACPs as monomeric species. Denatured ESMS confirmed the correct sequences. **A)** Y60F. **B)** D35S. **C)** E41A. **D)** E41R. **E)** R39A. **F)** D63A.

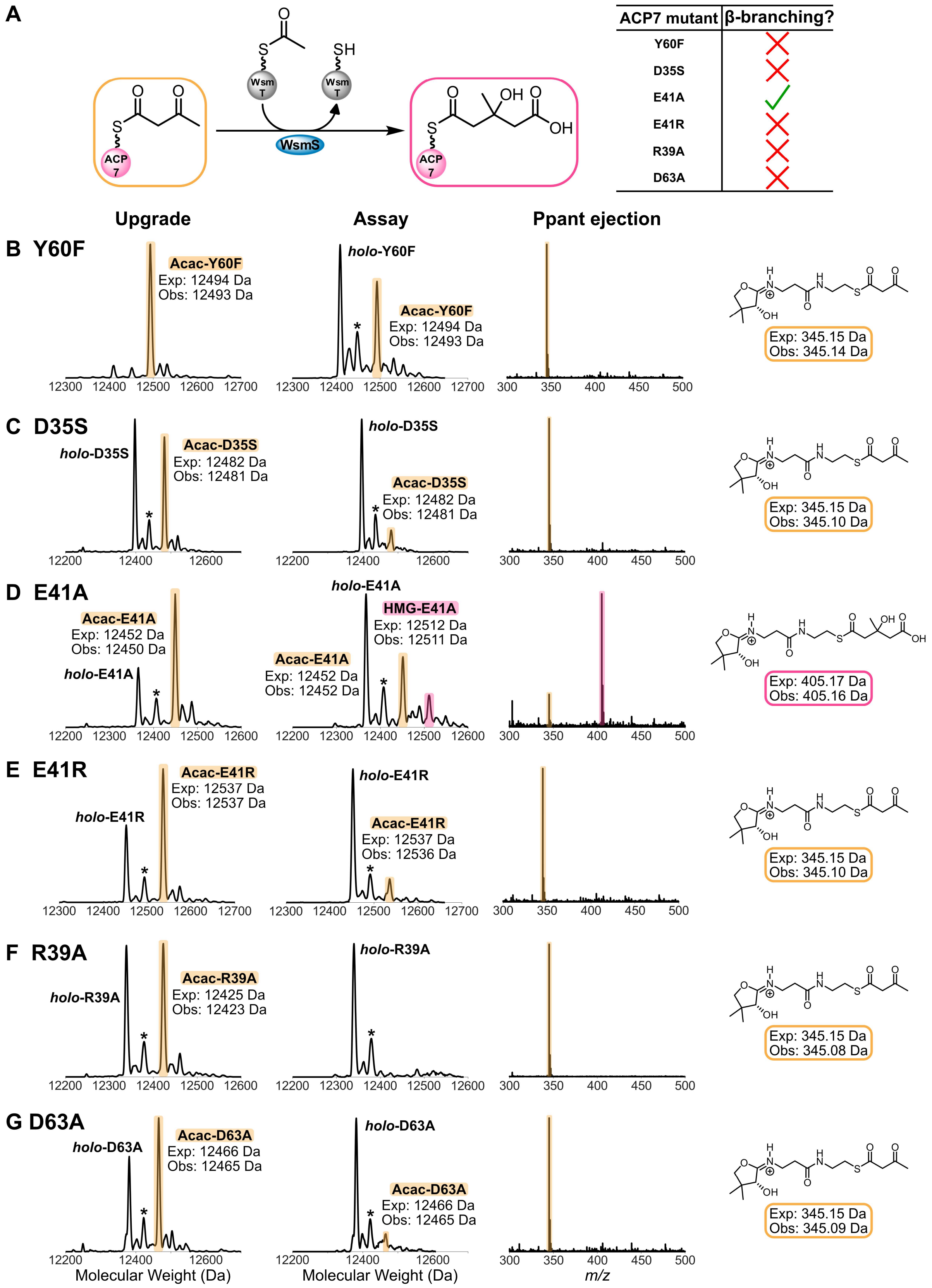

Supplementary Figure 18. WsmS ESMS assays with all WsmR ACP7 mutants. A) Reaction scheme for aldol addition of acetyl-WsmT with an acetoacetyl-WsmR ACP7 variant (yellow) catalyzed by WsmS to produce an HMG-WsmR ACP7 variant (pink). The table summarizes the results of the WsmS assays for each mutant. β-Branching was only observed for E41A. Deconvoluted MS of loaded-ACPs (left), corresponding WsmS assays (middle) and Ppant ejections (right) are shown for each WsmR ACP7 mutant as follows: **B)** Y60F. **C)** D35S. **D)** E41A. **E)** E41R. **F)** R39A. **G)** D63A.

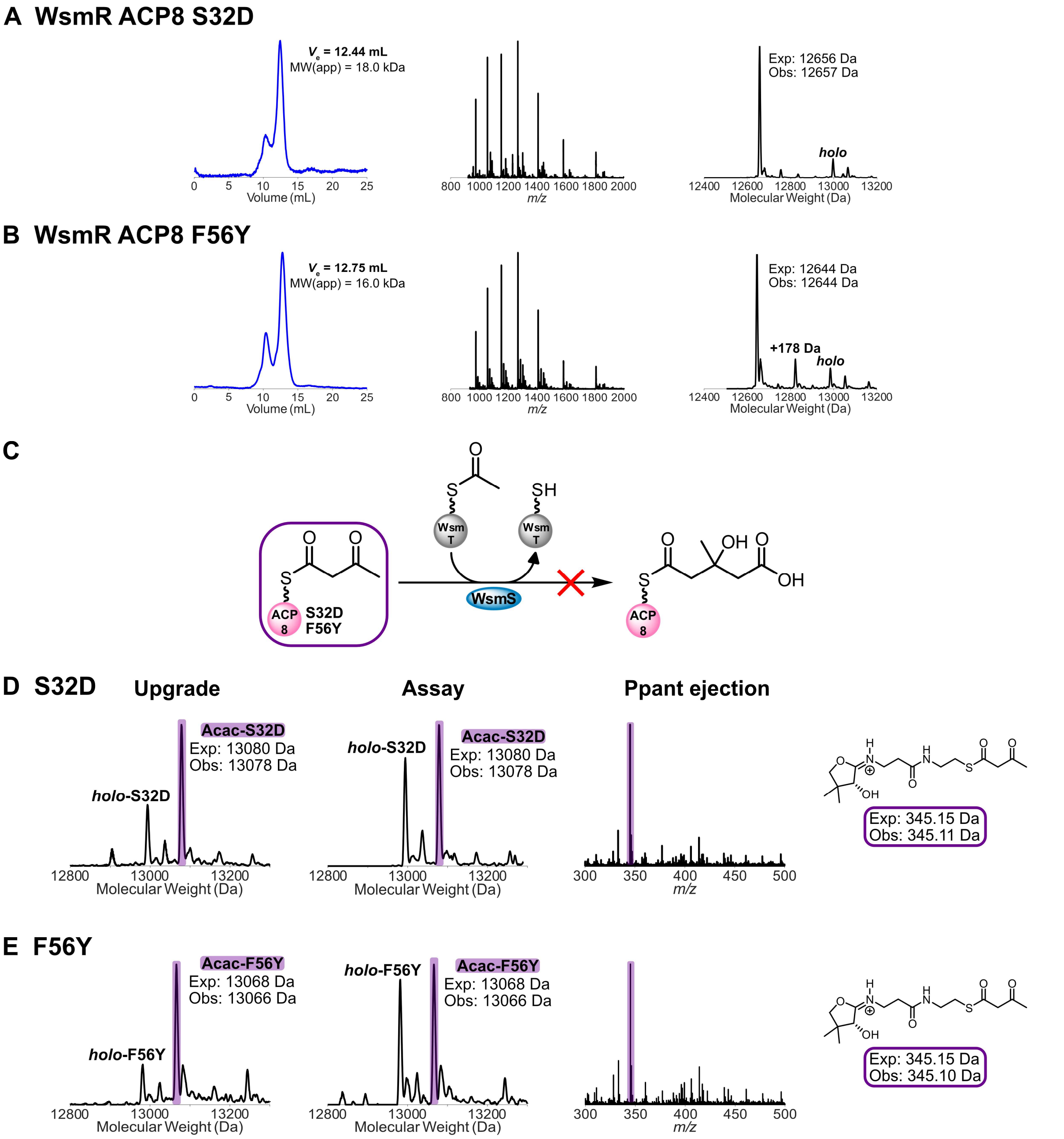

Supplementary Figure 19. Purification and characterization of WsmR ACP8 mutants and WsmS ESMS assays. A) Purification of WsmR ACP8 S32D. Analytical SEC showing elution mainly as a monomeric species. Denatured ESMS confirmed the correct sequence. **B)** Purification of WsmR ACP8 F56Y. Analytical SEC showing elution mainly as a monomeric species. Denatured ESMS confirmed the correct sequence. Approximately 15% was phosphogluconoylated (+178 Da) as observed by ESMS, resulting from post-translational modification of the His_6-tag.[10] **C)** Reaction scheme for WsmS-catalyzed aldol addition of acetyl-WsmT with acetoacetyl-WsmR ACP8 variants (purple) to produce an HMG-WsmR ACP8 variant. Neither of the ACP8 variants were able to act as an ACP_A. **D)** Deconvoluted MS of acetoacetyl-WsmR ACP8 S32D upgrade (left) and corresponding WsmS assay (middle). HMG-WsmR ACP8 S32D was not observed (exp: 13140 Da). Ppant ejection of acetoacetyl-WsmR ACP8 S32D (right). **E)** Deconvoluted MS of acetoacetyl-WsmR ACP8 F56Y upgrade (left) and corresponding WsmS assay (middle). HMG-WsmR ACP8 F56Y was not observed (exp: 13128 Da). Ppant ejection of acetoacetyl-WsmR ACP8 F56Y (right).

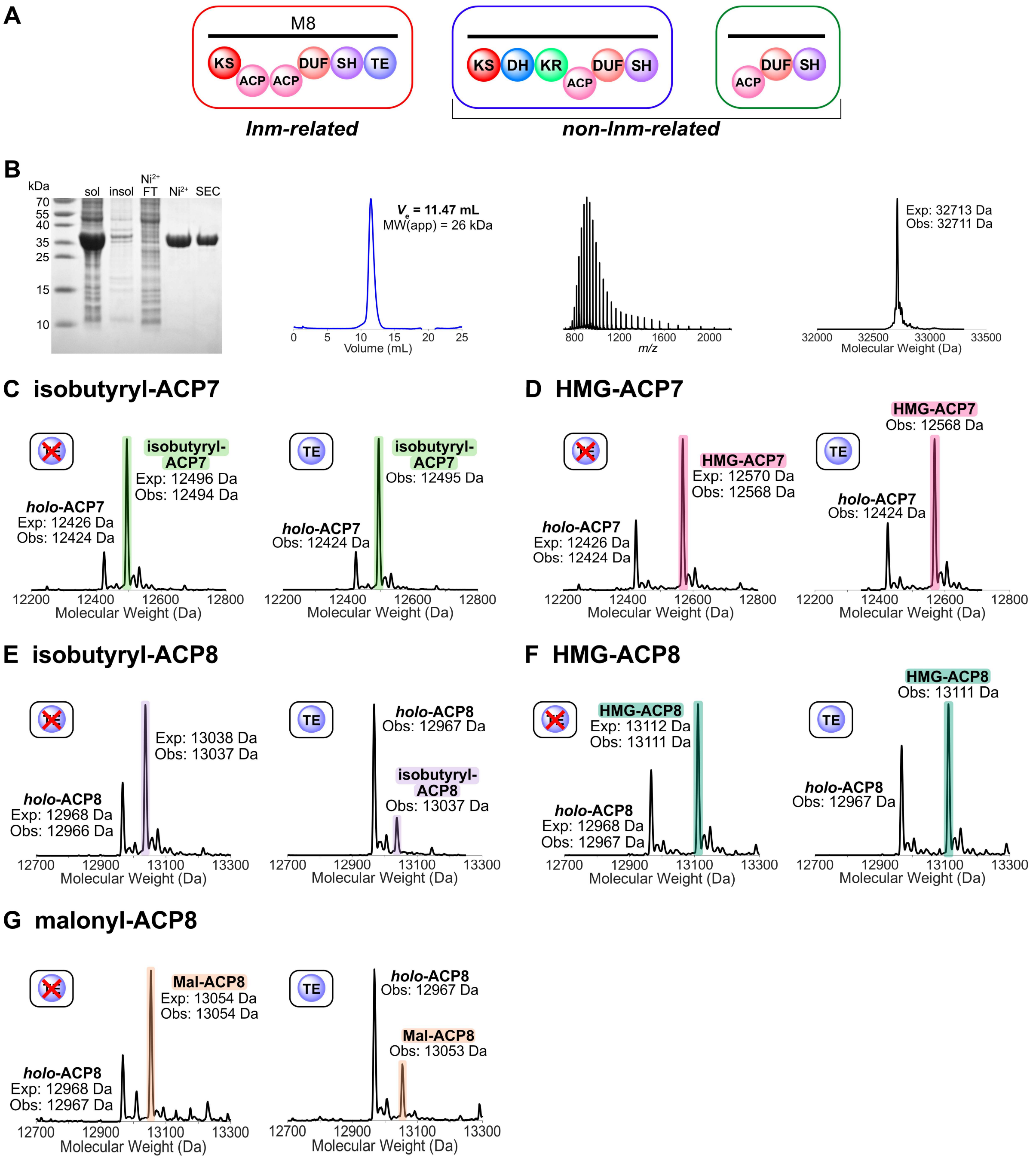

Supplementary Figure 20. WsmR TE purification and ESMS assays. A) Modules containing the DUF-SH didomain in leinamycin and non-leinamycin related PKSs.[6b] Only the leinamycin-related modules harbour two ACPs and a TE. **B)** Purification of WsmR TE. SDS-PAGE following purification by IMAC and SEC. Analytical SEC showing elution of WsmR TE as a monomeric species. ESMS of denatured WsmR TE. **C)** Isobutyryl-WsmR ACP7 in the absence (left) and presence (right) of WsmR TE. **D)** HMG-WsmR ACP7 in the absence (left) and presence (right) of WsmR TE. **E)** Isobutyryl-WsmR ACP8 in the absence (left) and presence (right) of WsmR TE. **F)** HMG-WsmR ACP8 in the absence (left) and presence (right) of WsmR TE. **G)** Malonyl (Mal)-WsmR ACP8 in the absence (left) and presence (right) of WsmR TE.

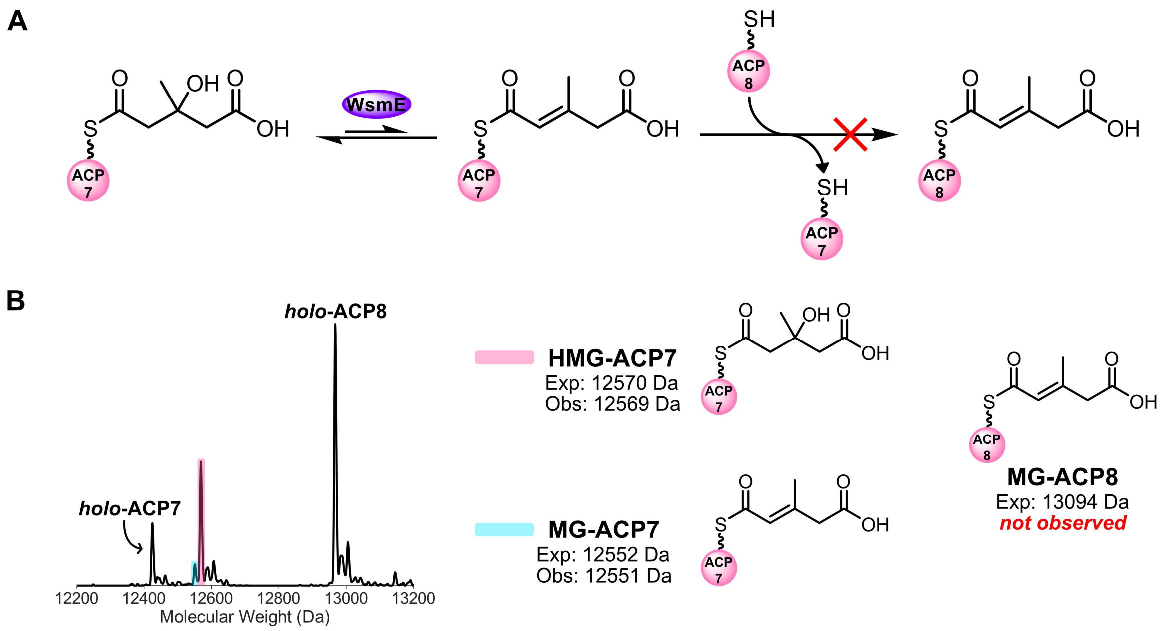

Supplementary Figure 21. Spontaneous transacylation assay. A) Proposed reaction scheme for spontaneous MG transfer from WsmR ACP7 to *holo*-WsmR ACP8. **B)** Deconvoluted MS of HMG-WsmR ACP7 and *holo*-WsmR ACP8 in the presence of WsmE. No spontaneous substrate transfer to *holo*-WsmR ACP8 was observed (exp: 13094 Da).

9. Amino acid residue sequences

WsmR ACP4

MHHHHHHGKPIPNPLLGLDSTENLYFQGIDPFTGPTGDDLRDEVLAVVAAQLYLAPHEVDTR
QTFSDMGLDSIGAVEVTNILATRLGVHLESVLVYERPTVPELADAVAEAVRHTRAIAEAAVA

Calculated Mw: 13432.11 Da

WsmR ACP6

MHHHHHHGKPIPNPLLGLDSTENLYFQGIDPFTPVADRIRAVEGDLRSLAAGFLMVSDEDVD
TATDLMELGFDSISLTELITRINARYGLDLLPTVLFETPTLEALAIRLANDHPQAGRPPAQASL

Calculated Mw: 13816.64 Da

WsmR ACP7

MHHHHHHGKPIPNPLLGLDSTENLYFQGIDPFTTGVTLADLRERVAGTLGLPVAEIGEQDSL
TGLGLDSIMRVELVRWLHESHGVTVPTSELYEHDTLAALARHLETAAAA

Calculated Mw: 12085.61 Da

WsmR ACP7 D35S

MHHHHHHGKPIPNPLLGLDSTENLYFQGIDPFTTGVTLADLRERVAGTLGLPVAEIGEQDSL
TGLGLSSIMRVELVRWLHESHGVTVPTSELYEHDTLAALARHLETAAAA

Calculated Mw: 12057.60 Da

WsmR ACP7 R39A

MHHHHHHGKPIPNPLLGLDSTENLYFQGIDPFTTGVTLADLRERVAGTLGLPVAEIGEQDSL
TGLGLDSIMAVELVRWLHESHGVTVPTSELYEHDTLAALARHLETAAAA

Calculated Mw: 12000.50 Da

WsmR ACP7 E41A

MHHHHHHGKPIPNPLLGLDSTENLYFQGIDPFTTGVTLADLRERVAGTLGLPVAEIGEQDSL
TGLGLDSIMRVALVRWLHESHGVTVPTSELYEHDTLAALARHLETAAAA

Calculated Mw: 12027.57 Da

WsmR ACP7 E41R

MHHHHHHGKPIPNPLLGLDSTENLYFQGIDPFTTGVTLADLRERVAGTLGLPVAEIGEQDSL
TGLGLDSIMRVRLVRWLHESHGVTVPTSELYEHDTLAALARHLETAAAA

Calculated Mw: 12112.68 Da

WsmR ACP7 Y60F

MHHHHHHGKPIPNPLLGLDSTENLYFQGIDPFTTGVTLADLRERVAGTLGLPVAEIGEQDSL
TGLGLDSIMRVELVRWLHESHGVTVPTSELFEHDTLAALARHLETAAAA

Calculated Mw: 12069.61 Da

WsmR ACP7 D63A

MHHHHHHGKPIPNPLLGLDSTENLYFQGIDPFTTGVTLADLRERVAGTLGLPVAEIGEQDSL
TGLGLDSIMRVELVRWLHESHGVTVPTSELYEHATLAALARHLETAAAA

Calculated Mw: 12041.60 Da

WsmR ACP8

MHHHHHHGKPIPNPLLGLDSTENLYFQGIDPFTAPEPAAVVRGAVERAVRTDLGPDGRFTD
GALSSLDMVRAVRALETVLGTLPKTLLFDQPDCTALAAHLTERFGAEAVARLADATA

Calculated Mw: 12628.31 Da

WsmR ACP8 S32D

MHHHHHHGKPIPNPLLGLDSTENLYFQGIDPFTAPEPAAVVRGAVERAVRTDLGPDGRFTD
GALDSLDMVRAVRALETVLGTLPKTLLFDQPDCTALAAHLTERFGAEAVARLADATA

Calculated Mw: 12656.32 Da

WsmR ACP8 F56Y

MHHHHHHGKPIPNPLLGLDSTENLYFQGIDPFTAPEPAAVVRGAVERAVRTDLGPDGRFTD
GALSSLDMVRAVRALETVLGTLPKTLLYDQPDCTALAAHLTERFGAEAVARLADATA

Calculated Mw: 12644.31 Da

His$_6$-WsmT

MHHHHHHGKPIPNPLLGLDSTENLYFQGIDPFTMDHTDGVVLAAIRAHATAILPDLAPADIVP
ERTLAELGLNSVDRSEVVTNVMDDLDVLVPITEFRQAMPVAELVVLFERYR

Calculated Mw: 12679.49 Da

WsmT (His$_6$-tag cleaved)

GIDPFTMDHTDGVVLAAIRAHATAILPDLAPADIVPERTLAELGLNSVDRSEVVTNVMDDLDV
LVPITEFRQAMPVAELVVLFERYR

Calculated Mw: 9526.95 Da

WsmD

MHHHHHHGKPIPNPLLGLDSTENLYFQGIDPFTMGVGIRALHPYVGRICVDIRTLYETRGHD
MSRFGNLMMSRKSVNMPFEDAVTNAVNAALPLVRRLSPAERDRIEAVVVATESGVDLGKSL
STYVHHYLELSPRCRSFEVKQACYGGTAALHTAVGMVHASPYDDALALVIAADSAGVPALG
EQWEGAEGAGAVAMLVGREATTLEIDLGAHGFHTFEVMDTFRPGPGIDVVNQDVSMLAYLE
CLQRAFTAYRDRVDGADIVDSFAYLAFHTPFVGMVRGAHRRLLRQQRQAGQHEVDSDYEQ
RVRPSQEYGAEVGNIFSASLYLALCSLIRHGSFPEPRRVGLFSYGSGCASEFFSGVIHPQAV
EDLAALGVEEALAARRPLTVAEYDSVAKDGDRAQFGVREAVFDLDEYGDLGKEFTGRGLLV
LDRIEGYHRTYRWS

Calculated Mw: 48612.85 Da

WsmE

MHHHHHHGKPIPNPLLGLDSTENLYFQGIDPFTMELMSAPTIGPDTGPAGAAGPAVLTDQD
GPVTRLVINRPHRQNSLTAEDVALLRDALERAAADPRTRAVVLEGSGGSLCTGMDLAELSA
DDDADAGGEFFDLLRRLTEIPVTVVAAVDGRAVGGGVGLAAACDLVIATERSSFSLPEALWG
LLPCNILPFLVRRIGFQRAYAMTLSTASVSAADARLSGLVDEVEGPHGQQLRRLLGRVTKLD
RPTVAEAKAYCAGLAPIPDTARAHAGEVFARLLASPVVRTRIDNFVHHQRMPWEG

Calculated Mw: 31985.40 Da

WsmR KS6

MHHHHHHGKPIPNPLLGLDSTENLYFQGIDPFTMPVAVIGMAGRFPGADDLAALWRVVSAG
EDRVGPVPADRAELLADPGMRDVRAGFLERVAEFDAAAFGISPREAGFMDPQQRQFLEVT
WQALYDAGRRPGELAGSATGVFVGVATGDYNELMAAHGGAPEAHMATGVAHAVLANRVS
HLLDLRGPSEAIDTACSSSLVAVHHAVRALQHGDCELAVAGGVNLTLSPALYTTFDRAGMLS
ARGRCAAFDDSADGYVRGEGVGAVVLKPLDRALADGDPVQAVILGTAVNHTGRTPSLTAPN
PHSQAEVIVEAVRAAGIDPRTIGFVQAHGTGTPLGDPVEIEGLKQAFARLYEDHDLPAPAEPH
LAVGTVKANIGHLEAAAGIAGLLTTVLAMRHGLIPPHPHLSEPNRYLRLDGTPLTLAHRARAW
EPTADESARPVRRAGVSSFGFGGSNAHVVLQTGGAAPARRPAAQGPLVVPLSARDGAALA
DYRLRLADALDALPDAGLDQVAYTLQVGREELPHRFAVVAADRTRLVAALRGTDQGGVHLG
DGTARPGGDASPVTPEELAAAWCAGRSVGWAGLWSAAPGRIPLPGPGFARTAYWYPRAA
A

Calculated Mw: 63599.79 Da

WsmS

MHHHHHHGKPIPNPLLGLDSTENLYFQGIDPFTMSSGPVTVGVEALDVYGGSAWISADDLA
EGRGLDRARVKNLMLSGRSVALPFEDPVTHAVNAAKGLLDDLDPAVRDRIEVVLVATESGVD
YSKSIASYVHRHLELPERCRLLEVKQACYAATAALQLAAGYLASGASPGARVLLIATDVGLAD
ERAEYAELATGSGAVALLLSERPDVLALDLGAFGLHSFETMDTARPGPDTEFADADLSLVAY
LECTVRSFADYSRRVADVRLDETFDHLVFHTPFAGMVRAAHRRAMREFTGLDPAATAADFT
RRVEPGLRYPAQVGNLFSGSLYLALAGLVDELPATARESRVGLFSYGSGCSSEFLSGTLGET
ARDTVAARKIGPRLAARRRLGFAEYRELLPYGQACLLPRRHLTLDPHPEWRPAAGGRGPLL
ALRGIDDYHRRYDWI

Calculated Mw: 48468.87 Da

WsmR TE

MHHHHHHGKPIPNPLLGLDSTENLYFQGIDPFTAGENMAVLTEHVPAVRRHLVELEDATVEA
FVHGTGPTLLLAHPFNIGAGMFARQFADLGTDHRLVVLHHPGVGATRTGGALSLDHIVDLYT
ATLTRLGIDGPVHLVGASWGALVAETFALRHPGRSASLTTVGGSYRYANRVGEVNRLEVIVA
EDMAAVAAATGDGARLPGRAAQLLRCESMDAYVGLSYLDLFAGEPDLLRRLPELTGMPTAVI
HGRLDSVVPLDTAHRLAAALPGARYEELADAGHFPCVTHADDVSRVLREVVAAATADRP

Calculated Mw: 32713.15 Da

LnmJ ACP8

MHHHHHHGKPIPNPLLGLDSTENLYFQGIDPFTPEAAAASTVVGLLRGELSKILGMPSEEIEN
DAPFGELGLDSIYRMDLVRTLNEAFGLDLKATELYNYDTIGKLTEFVAPLVG

Calculated Mw: 12614.30 Da

LnmJ ACP8-9

MHHHHHHGKPIPNPLLGLDSTENLYFQGIDPFTPEAAAASTVVGLLRGELSKILGMPSEEIE
NDAPFGELGLDSIYRMDLVRTLNEAFGLDLKATELYNYDTIGKLTEFVAPLVGPAGAAPAAE
PVMAEAPQQSSASLEDLVQDVIERELGRTADPAKSFVDNGFGSFDMLRVVASLERVFGAL
RKTLLFDHPTIGALAAHLAETHGPEAAS

Calculated Mw: 22707.64 Da

LnmJ ACP8-9 S41A

MHHHHHHGKPIPNPLLGLDSTENLYFQGIDPFTPEAAAASTVVGLLRGELSKILGMPSEEIE
NDAPFGELGLDAIYRMDLVRTLNEAFGLDLKATELYNYDTIGKLTEFVAPLVGPAGAAPAAE
PVMAEAPQQSSASLEDLVQDVIERELGRTADPAKSFVDNGFGSFDMLRVVASLERVFGAL
RKTLLFDHPTIGALAAHLAETHGPEAAS

Calculated Mw: 22691.64 Da

LnmF

MHHHHHHGKPIPNPLLGLDSTENLYFQGIDPFTMTAIGPTHRGVRLTAEPHVLRATLTSPDG
LNSLSGAALDALGAALDRAEADPECRVLLLEGSGGTFCTGLDFEEAAGDPAGGASQAGRG
GAEFLALMRRFGETPLAVVACVDGRAAGGGVGLAAAADLVIATERSEFSLPEALWGLVPCC
VLPVLVRRTGFQPAYAMALSTQPVSARRAADFRLVDEVVPDPDAAVRRLLVRLTRLDPATI
GELKQYFRAMWFTTEDTDAFALREFTRLIDSPVARRRITDYTTTRRLPWEKPRP

Calculated Mw: 32086.60 Da

LnmG

MHHHHHHGKPIPNPLLGLDSTENLYFQGIDPFTMVALVFPGQGSQRKGMGADLFARFPDL
TRQADTVLGHSVEELCRSSGDGRLDRTEYAQPALFVVSALSYLARDPGLPQPTLLAGHSLG
EYGALFAAGCFDFATGVRLVRERGALMGRAQGGGMLAVLGVDGDEVQALLAGTGARQVD
VANYNTPTQTVLSGPLDELRMVSAALGQRPGVRCVPVRVSAAFHSRHMRPAAQEFATFLT
GFSFADPHRTVISSVTARPYGAGQVAELLSRQIESPVRWSETMAYLRERGTTELEEMGPGK
VLTGLWKQGRADGAKARAVAPAPVAVVAGVPAAAAARAPASPAPVAARAATAAPARPSDP
APPAPRTAPSPASPVPPASVSRGQRAEELGSAEFRQDYGIRYAYLAGAMFRGIASAELVIR
MGRAGLMGFFGAGGLGLDKVESALVRIKDALGPDGRYGMNLLHSIDDPAYEHAVVDLCLK
HGVHDVEAAGFTQLTPAVVQFRFSGAHRDAAGRAVAVRRVLAKVSRPEVAAAFMAPAPAA
ILRRLTADGRLTPQEAEIAAELPVGQDICVEADSGGHTDGGAALTLLPSMIRHRDAAMARHG
YGRRIRIGAAGGIGAPEAVAAAFVLGADFVLTGSVNQCSPEAGTSDAVKDILAGLDVQDTAY
APAGDMFEIGARVQVVRKGTLFAARGNKLYQLYRSHDSWESIDAGTRRSVEETYFKRPFA
EVWEETRAYHLGRGRDAEIEKADRLPKHRMALAFRWYFARSVRWGLEGEPTQKVNYQIQ
CGPAIGAFNHVVRGTGLEDWRHRHVDLIAEHLMTGAADVLARR

Calculated Mw: 88210.28 Da

**Primer**	**Sequence (5'-3')**
LnmJ_ACP8-9_A41S_F	GAATTAGGTCTGGAT**AGC**ATTTATCGTA
LnmJ_ACP8-9_A41S_R	AAATGATGCACCGTTTGGT
WsmR_ACP7_D35S_F	TCTGGGTTTA**AGC**AGCATTATGCGT
WsmR_ACP7_D35S_R	CCGGTCAGGCTATCCTGTTCA
WsmR_ACP7_R39A_F	GATAGCATTATG**GCA**GTTGAACTGGTT
WsmR_ACP7_R39A_R	TAAACCCAGACCGGTCAGGC
WsmR_ACP7_E41A_F	ATTATGCGTGTT**GCA**CTGGTTCGTT
WsmR_ACP7_E41A_R	GCTATCTAAACCCAGACCGGTCAG
WsmR_ACP7_E41R_F	ATTATGCGTGTT**CGT**CTGGTTCGTT
WsmR_ACP7_E41R_R	GCTATCTAAACCCAGACCGGTCAG
WsmR_ACP7_Y60F_F	GACCAGCGAACTG**TTT**GAACATGATACC
WsmR_ACP7_Y60F_R	GGAACGGTAACACCATGGCTTTCA
WsmR_ACP7_D63A_F	CTGTATGAACAT**GCA**ACCCTGGCA
WsmR_ACP7_D63A_R	TTCGCTGGTCGGAACGGTAAC
WsmR_ACP8_S32D_F	ATGGTGCACTG**GAT**AGCCTGGATATG
WsmR_ACP8_S32D_R	CGGTAAAACGACCATCAGGACCC
WsmR_ACP8_F56Y_F	CGAAAACACTGCTG**TAT**GATCAGCCG
WsmR_ACP8_F56Y_R	GCAGGGTGCCCAGAACGG

Table S1: A list of primers used in this study. Codons in bold mark the point of the mutation.

PKS	NCBI Accession Number
BuboR	NZ_LOTL00000000
CaciV	NC_013131
CB01201T	NNBJ00000000
CB01373U	NNBK00000000
CB01635J	NNBL00000000
CB02613T	NNBN00000000
CB02891P	NNBO00000000
CB02959I	NNBP00000000
GnmT	LIWA00000000
LnmJ	AF484556
LrgJ	LR131959
MaurJ	NC_014391
McnbJ	NZ_ARGW00000000
MgloJ	NZ_JNZR00000000
Ml5J	NC_014815
MmarJ	NZ_FMCV00000000
MtuL	NZ_FMCQ00000000
S109L	NZ_BBON00000000
S110T	NZ_BBNN00000000
SaesK	NC_019673
SastY	NZ_JYJF00000000
SaurT	NZ_JODU00000000
ScanQ	NZ_LQCG00000000
Sf56T	NZ_JOGO01000001
ShygL	NZ_BBOU00000000
SleeL	NZ_AZSD01000122
SnovY	NZ_JNWQ00000000
TSRI0384L	NOWW00000000
WsmR	NNBM00000000

Table S2. NCBI accession numbers for the genomes/BGCs encoding all leinamycin-type PKSs used in phylogenetic analysis (Figure S2). Genomes/BGCs were analysed by antiSMASH and ACP amino acid sequences manually extracted.[3]

10. References

[1] a A. S. Haines, X. Dong, Z. Song, R. Farmer, C. Williams, J. Hothersall, E. Płoskoń, P. Wattana-Amorn, E. R. Stephens, E. Yamada, *Nat. Chem. Biol.* **2013**, *9*, 685-692; b V. Agarwal, S. Diethelm, L. Ray, N. Garg, T. Awakawa, P. C. Dorrestein, B. S. Moore, *Org. Lett.* **2015**, *17*, 4452-4455; c Y.-Q. Cheng, G.-L. Tang, B. Shen, *Proc. Natl. Acad. Sci. USA* **2003**, *100*, 3149-3154.

[2] P. D. Walker, C. Williams, A. N. Weir, L. Wang, J. Crosby, P. R. Race, T. J. Simpson, C. L. Willis, M. P. Crump, *Angew. Chem., Int. Ed.* **2019**, *58*, 12446-12450.

[3] K. Blin, S. Shaw, H. E. Augustijn, Z. L. Reitz, F. Biermann, M. Alanjary, A. Fetter, B. R. Terlouw, W. W. Metcalf, E. J. Helfrich, *Nucleic Acids Res.* **2023**, *51*, W46-W50.

[4] R. C. Edgar, *Nucleic Acids Res.* **2004**, *32*, 1792-1797.

[5] K. Tamura, G. Stecher, S. Kumar, *Mol. Biol. Evol.* **2021**, *38*, 3022-3027.

[6] a G. Pan, Z. Xu, Z. Guo, Hindra, M. Ma, D. Yang, H. Zhou, Y. Gansemans, X. Zhu, Y. Huang, *Proc. Natl. Acad. Sci. USA* **2017**, *114*, E11131-E11140; b S. Meng, A. D. Steele, W. Yan, G. Pan, E. Kalkreuter, Y.-C. Liu, Z. Xu, B. Shen, *Nat. Commun.* **2021**, *12*, 5672.

[7] A. Nivina, K. P. Yuet, J. Hsu, C. Khosla, *Chem. Rev.* **2019**, *119*, 12524-12547.

[8] A. Becerril, I. Perez-Victoria, S. Ye, A. F. Brana, J. Martin, F. Reyes, J. A. Salas, C. Mendez, *ACS Chem. Biol.* **2020**, *15*, 1541-1553.

[9] J. Jumper, R. Evans, A. Pritzel, T. Green, M. Figurnov, O. Ronneberger, K. Tunyasuvunakool, R. Bates, A. Žídek, A. Potapenko, *Nature* **2021**, *596*, 583-589.

[10] K. F. Geoghegan, H. B. Dixon, P. J. Rosner, L. R. Hoth, A. J. Lanzetti, K. A. Borzilleri, E. S. Marr, L. H. Pezzullo, L. B. Martin, P. K. LeMotte, *Anal. Biochem.* **1999**, *267*, 169-184.
